# Supplementary material for: Chemical Diversity of Mo5S5 Clusters with Pyrazole: Synthesis, Redox and UV-vis-NIR Absorption Properties
Source: Int J Mol Sci. 2023 Sep 9;24(18):13879. doi: 10.3390/ijms241813879 (PMC10531228; doi:10.3390/ijms241813879)
Supplement: Supplementary file 1 [file ijms-24-13879-s001.zip › ijms-2580047-supplementary.pdf]

Chemical diversity of Mo_5S_5 clusters with pyrazole: synthesis, redox and UV-vis-NIR absorption properties

Iulia V. Savina,[1] Anton A. Ivanov,[1] Ilia V. Eltsov,[2] Vadim V. Yanshole,[2,3] Natalia V. Kuratieva,[1] Andrey Y. Komarovskikh,[1] Mikhail M. Syrokvashin,[1] and Michael A. Shestopalov[1,*]

[1] Nikolaev Institute of Inorganic Chemistry of Siberian Branch of Russian Academy of Sciences, 3 Acad. Lavrentiev ave., 630090, Novosibirsk, Russia; savina@niic.nsc.ru (I.V.S.); ivanov338@niic.nsc.ru (A.A.I); kuratieva@gmail.com (N.V.K); komarovskikh@niic.nsc.ru (A.Y.K.); syrokvashin@niic.nsc.ru (M.M.S.); shtopy@niic.nsc.ru (M.A.S.)

[2] Novosibirsk State University, 1 Pirogova st., 630090, Novosibirsk, Russia; eiv@fen.nsu.ru (I.V.E.); Vadim.Yanshole@tomo.nsc.ru (V.V.Y.)

[3] International Tomography Center SB RAS, 3a Institutskaya Str., 630090 Novosibirsk, Russia; Vadim.Yanshole@tomo.nsc.ru (V.V.Y.)

* Correspondence: shtopy@niic.nsc.ru (M.A.S.)

TABLE OF CONTENT

NMR spectroscopy data for [1^{ox}]Br_2 3
Mass-spectrometry data for [1^{ox}]Br_2 6
NMR spectroscopy data for [2]Br 7
NMR spectroscopy data for [3]Br_2 10
Mass-spectrometry data for [2]Br and [3]Br_2 11
NMR spectroscopy data for [4]Br 12
Mass-spectrometry data for [4]Br 16
Packing in the crystal structures 17
EPR and DFT calculations data for [1^{red}]Br 28
Cyclic voltammetry data 29
DFT calculations data 34
UV-vis-NIR absorption data 35
IR spectra 36
Thermogravimetric analysis 37
X-ray powder diffraction analysis of reaction mixture 39
Crystal structure data 40

NMR spectroscopy data for [1^{ox}]Br$_2$

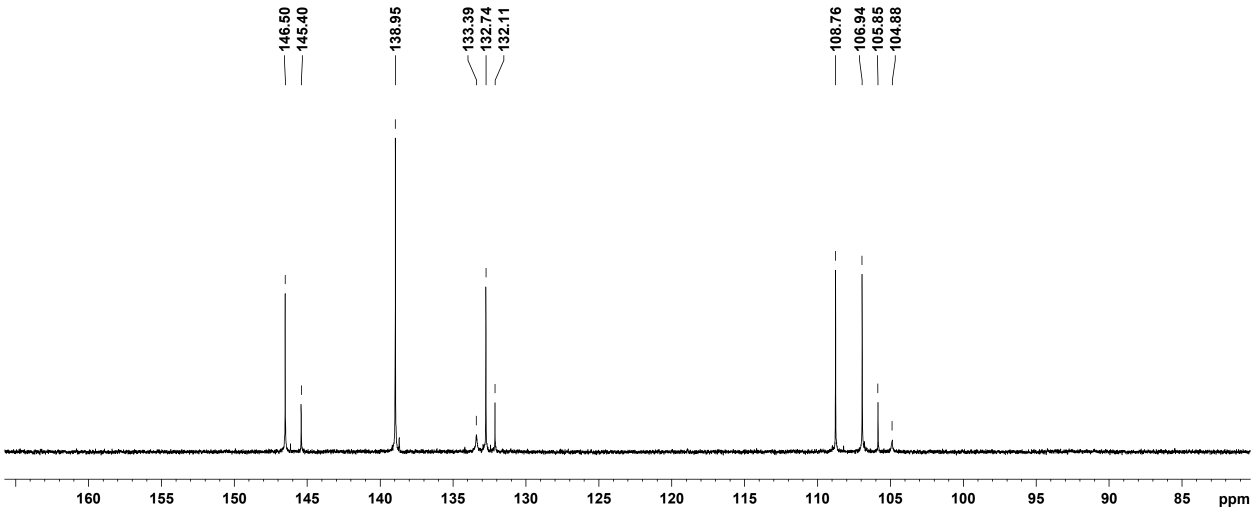

Figure S1. ^{13}C NMR spectrum of **[1^{ox}]Br$_2$** in CD_3OD.

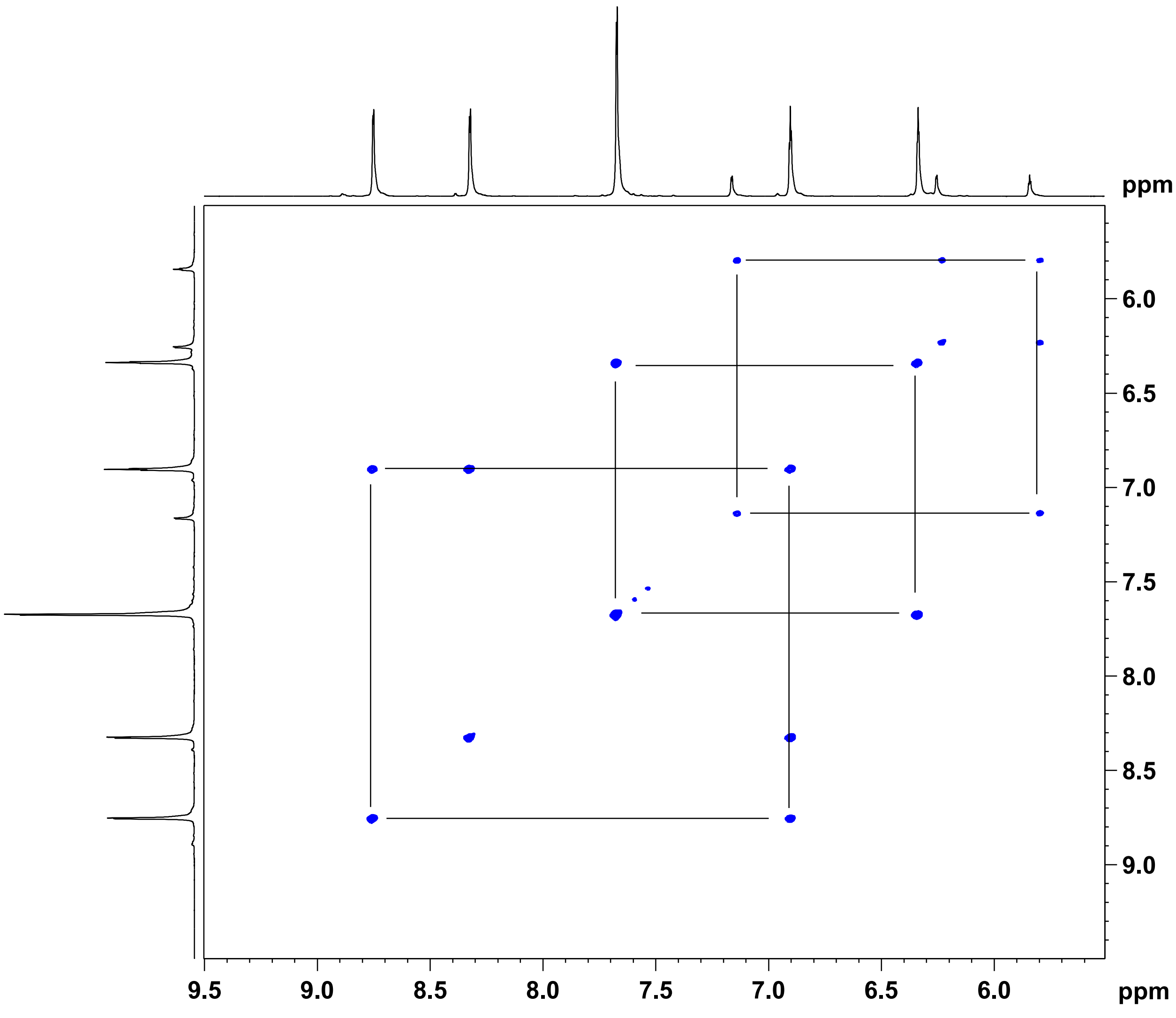

Figure S2. ^{1}H,^{1}H-NMR correlation spectra of **[1^{ox}]Br$_2$** in CD_3OD.

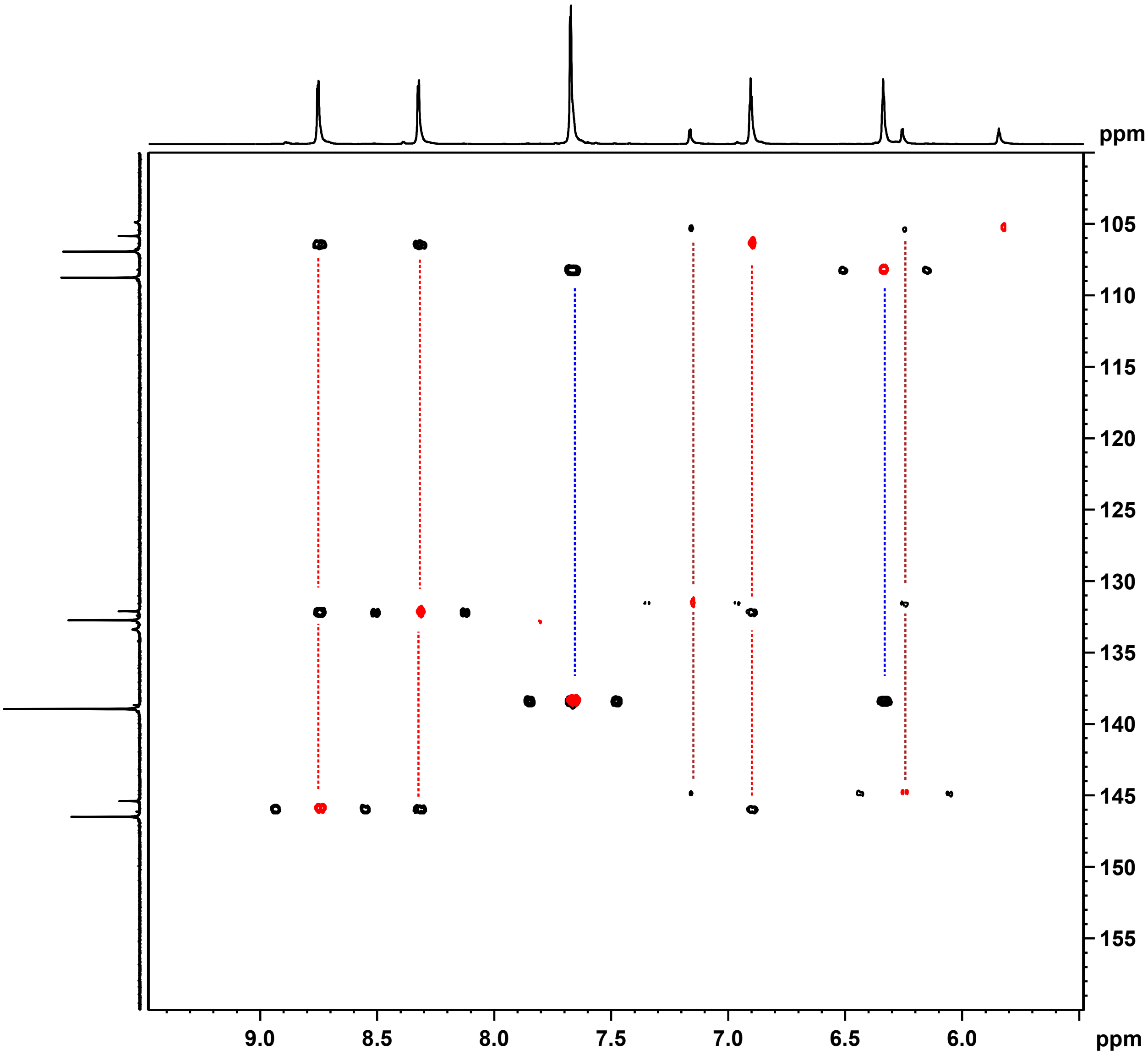

Figure S3. $^{1}H,^{13}C$-NMR correlation spectra (HMBC – black, HSQC – red) of **[1^{ox}]Br$_2$** in CD_3OD.

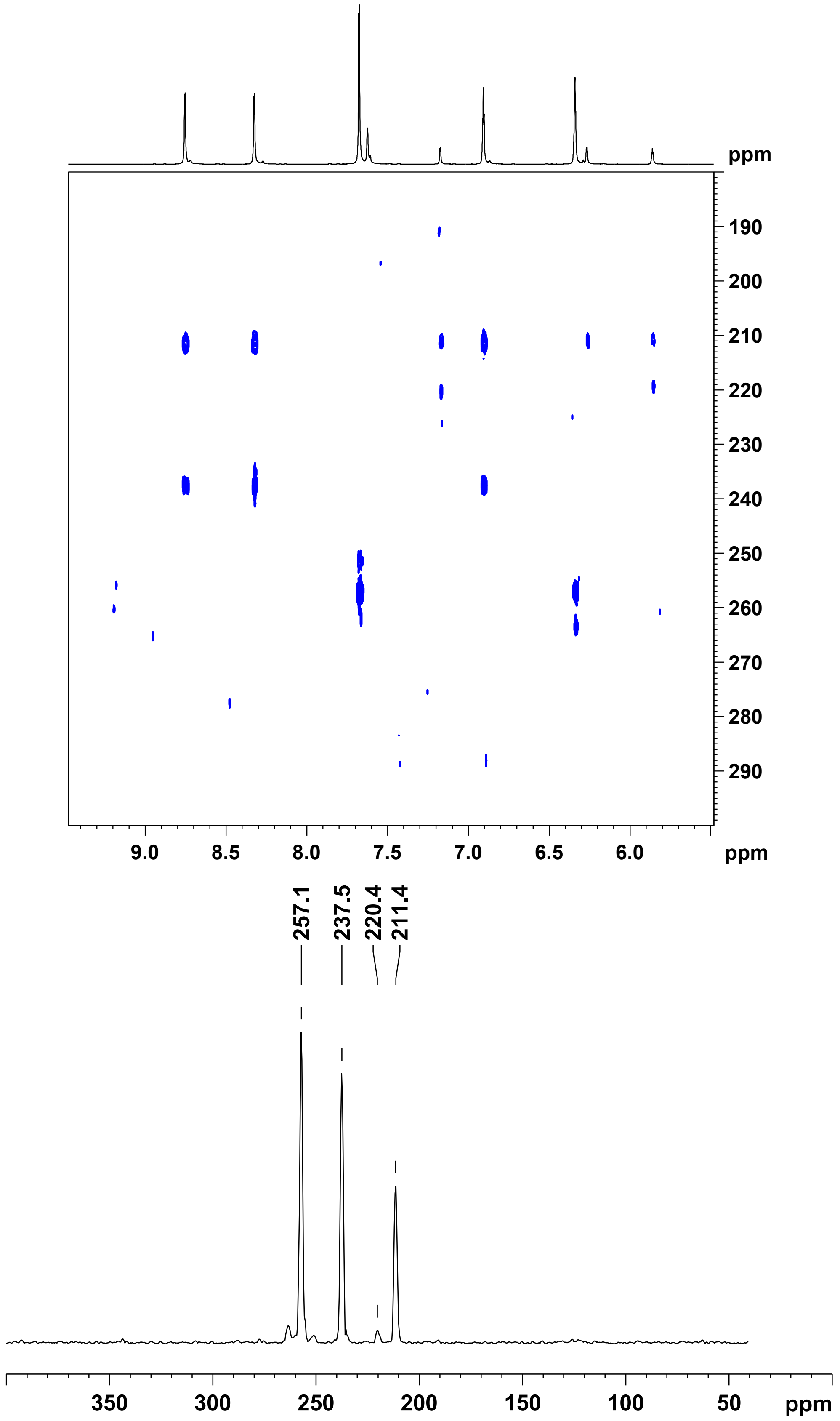

Figure S4. ^{1}H-^{15}N HMBC NMR spectra (top) and ^{15}N-axe projection (bottom) of **[1^{ox}]Br$_2$** in CD_3OD.

Mass-spectrometry data for [1^{ox}]Br_2

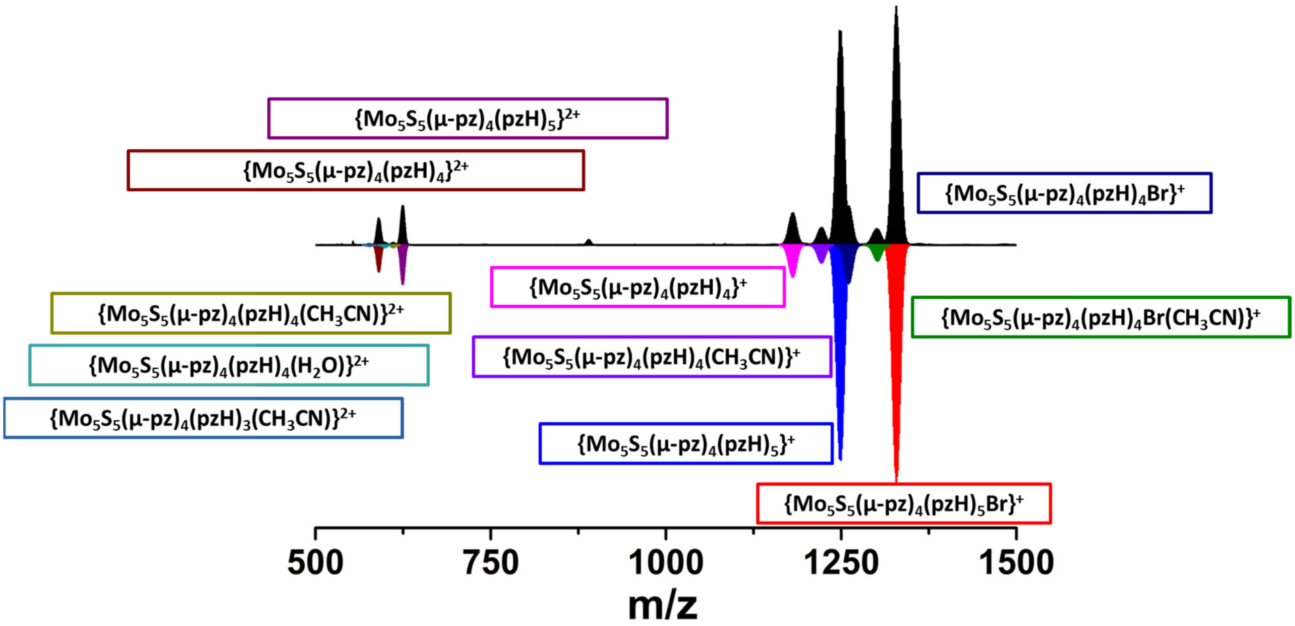

Figure S5. HR-ESI-MS spectrum of solution of **[1^{ox}]Br_2** in acetonitrile (black) and simulations of cluster forms (colored).

NMR spectroscopy data for [2]Br

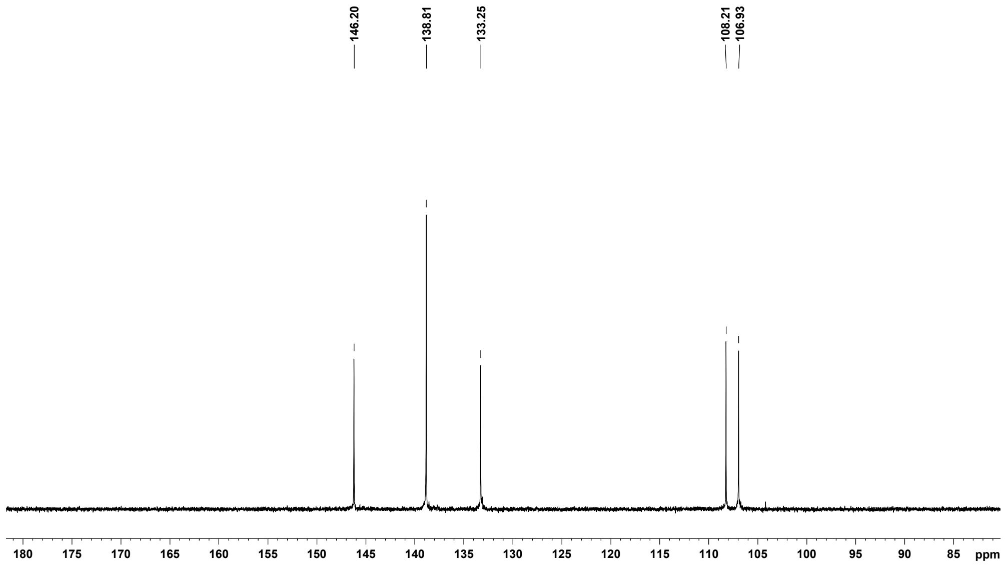

Figure S6. ^{13}C NMR spectrum of **[2]Br** in DMSO-d_6.

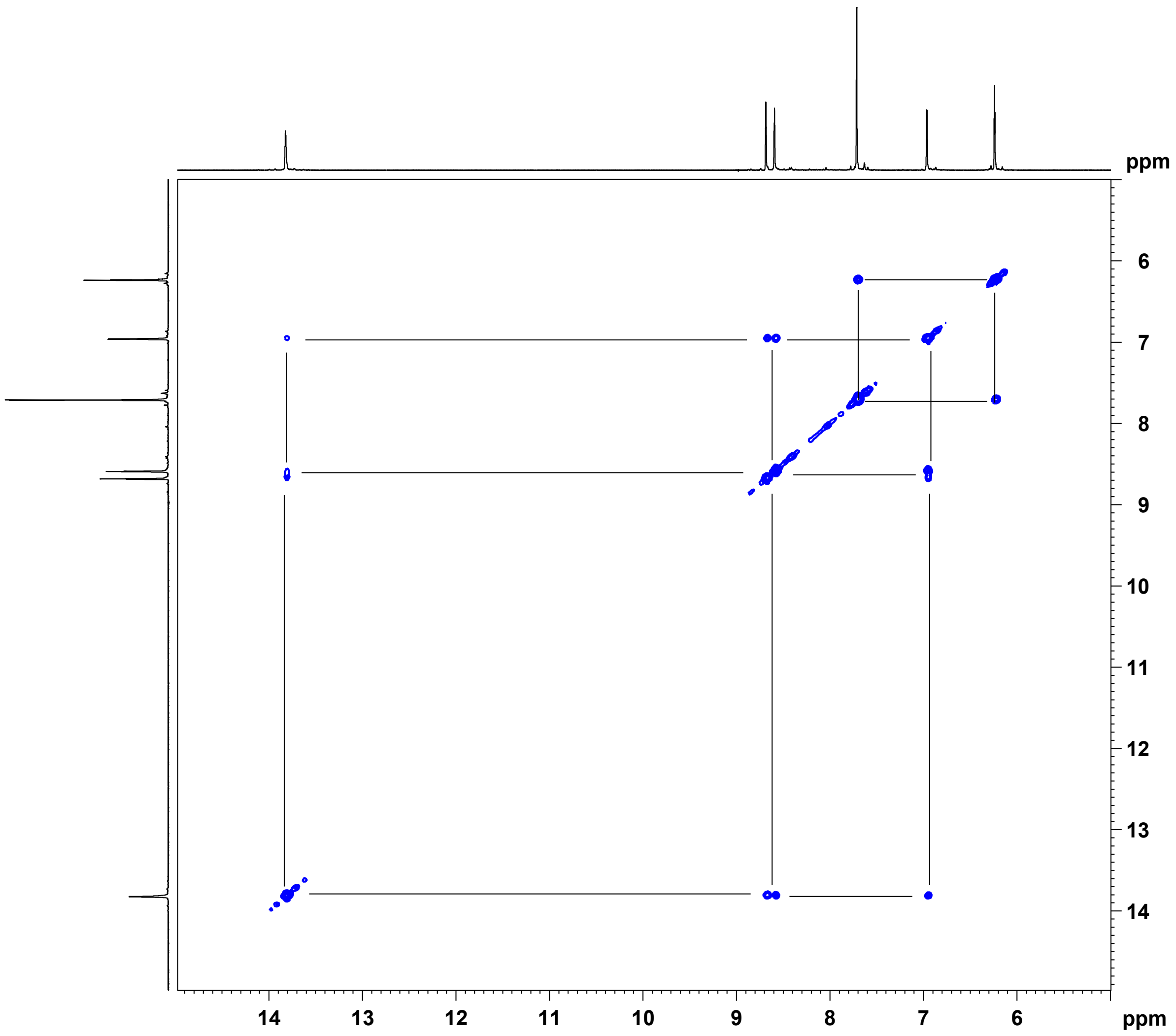

Figure S7. $^{1}H,^{1}H$-NMR correlation spectra of **[2]Br** in DMSO-d_6.

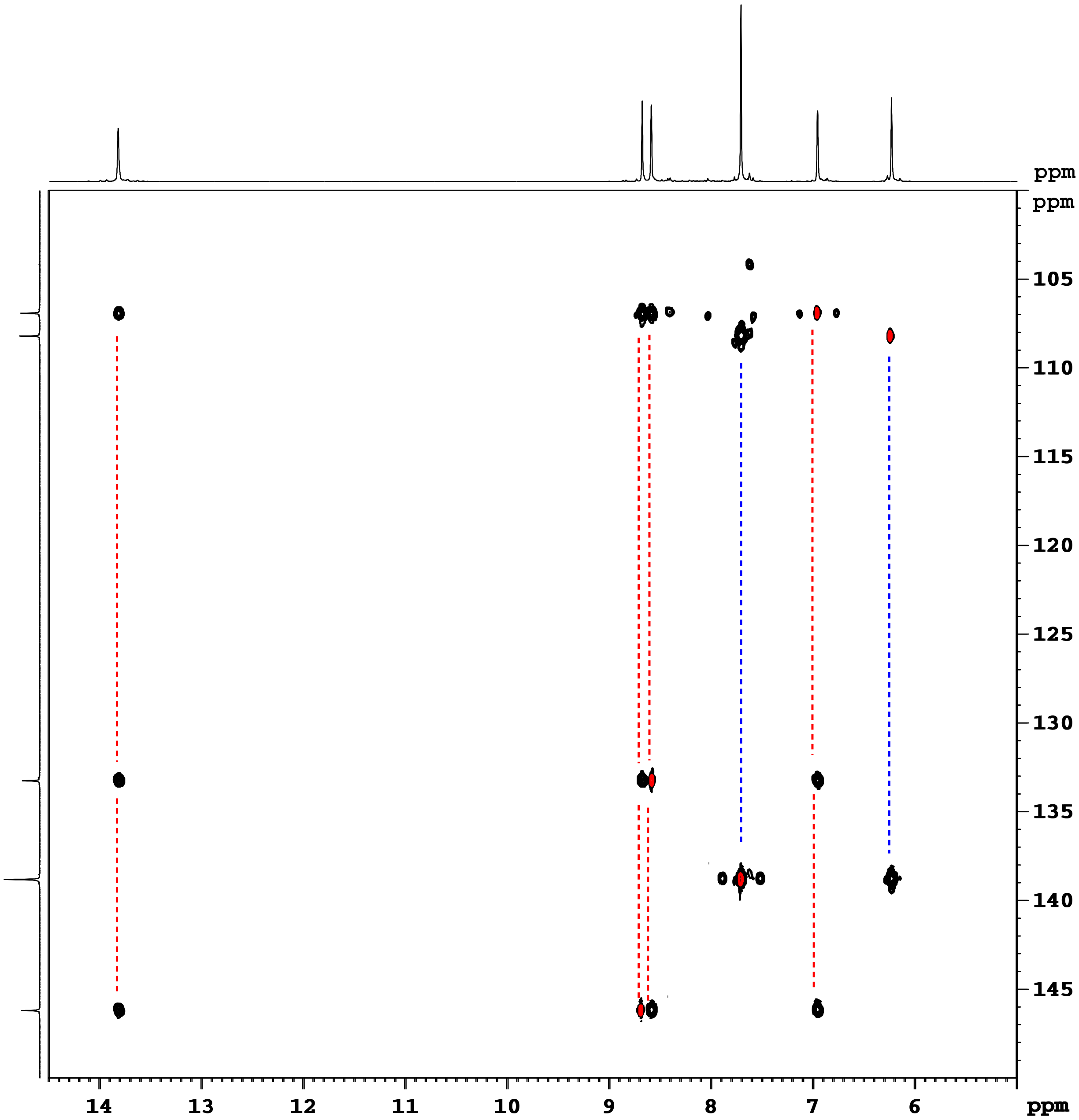

Figure S8. ^{1}H,^{13}C-NMR correlation spectra (HMBC – black, HSQC – red) of **[2]Br** in DMSO-d_6.

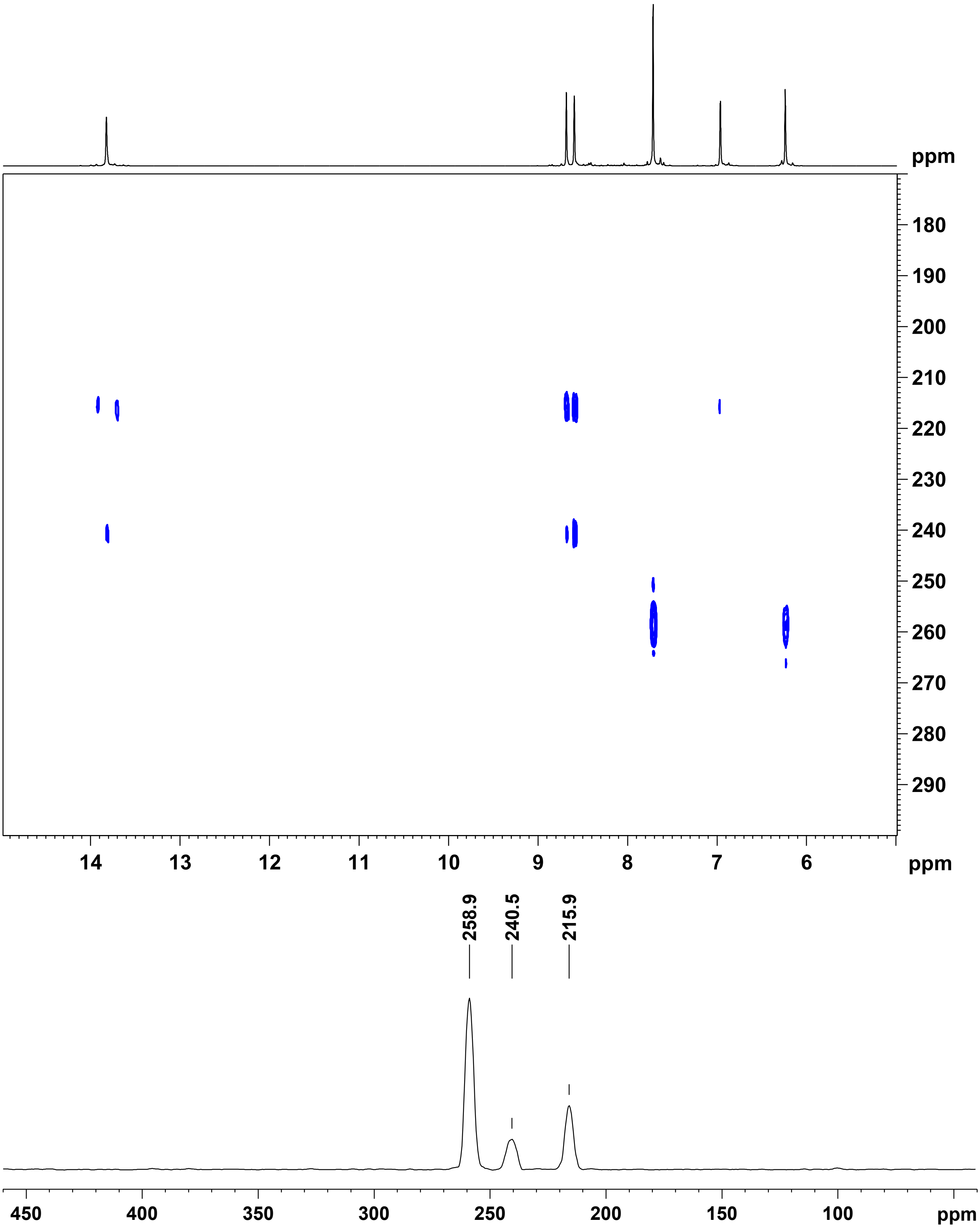

Figure S9. ^{1}H-^{15}N HMBC NMR spectra (top) and ^{15}N-axe projection (bottom) of **[2]Br** in DMSO-d_6.

NMR spectroscopy data for [3]Br_2

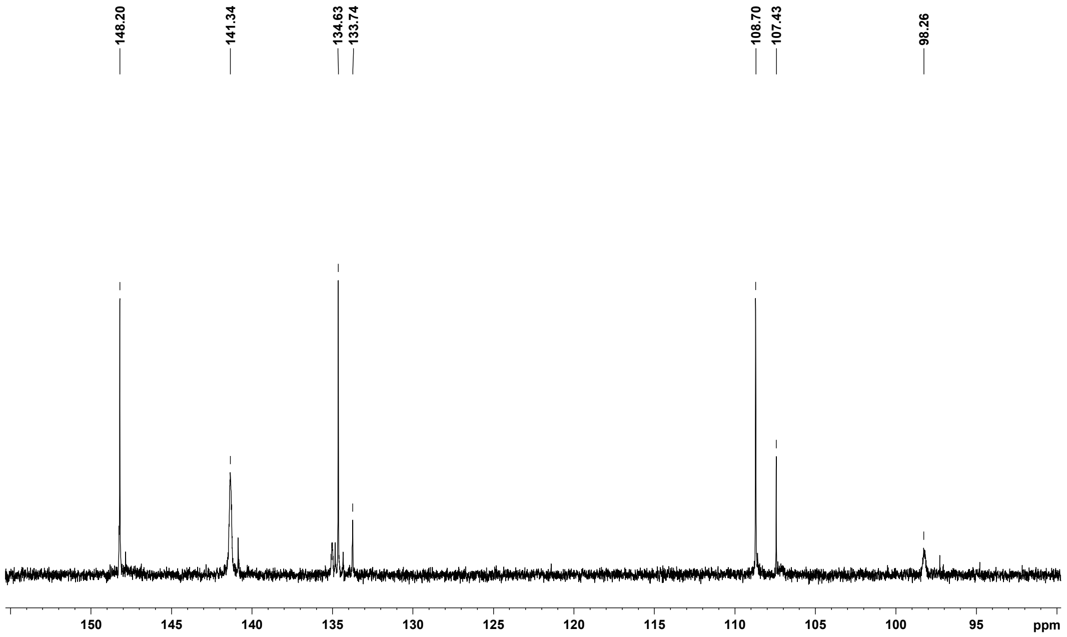

Figure S10. ^{13}C NMR spectrum of **[3]Br_2** in CD_3OD.

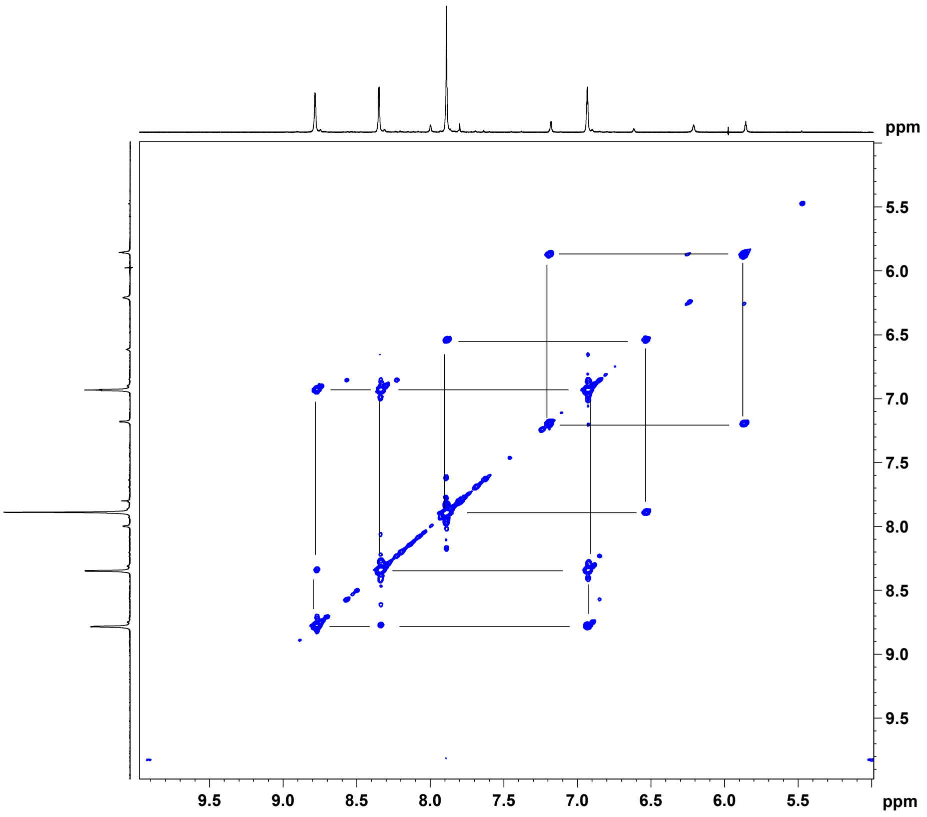

Figure S11. $^{1}H,^{1}H$-NMR correlation spectra of **[3]Br_2** in CD_3OD.

Mass-spectrometry data for [2]Br and [3]Br$_2$

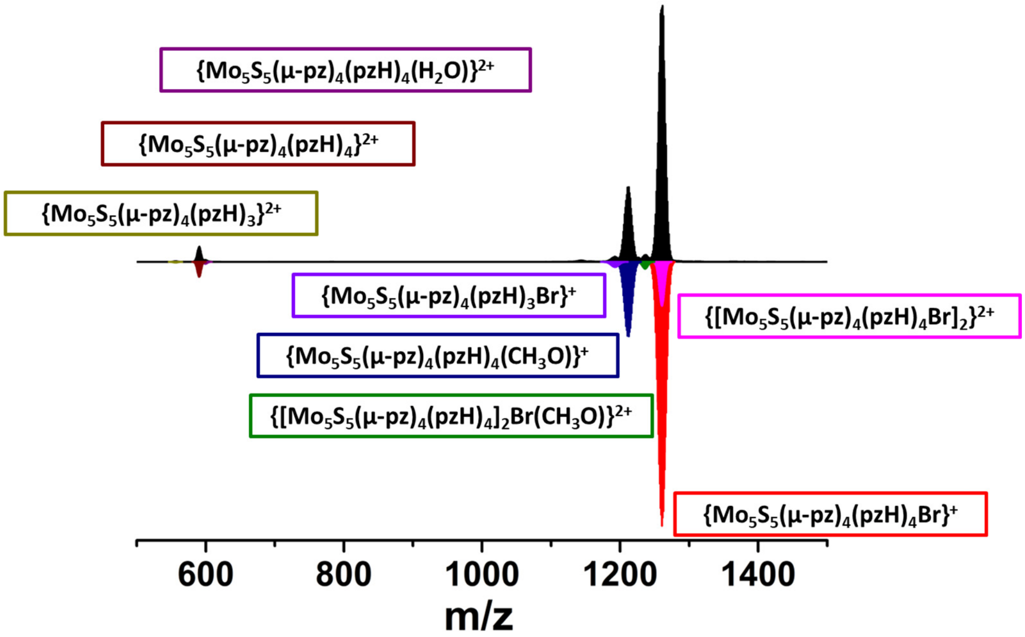

Figure S12. HR-ESI-MS spectrum of solution of **[2]Br** in methanol (black) and simulations of cluster forms (colored).

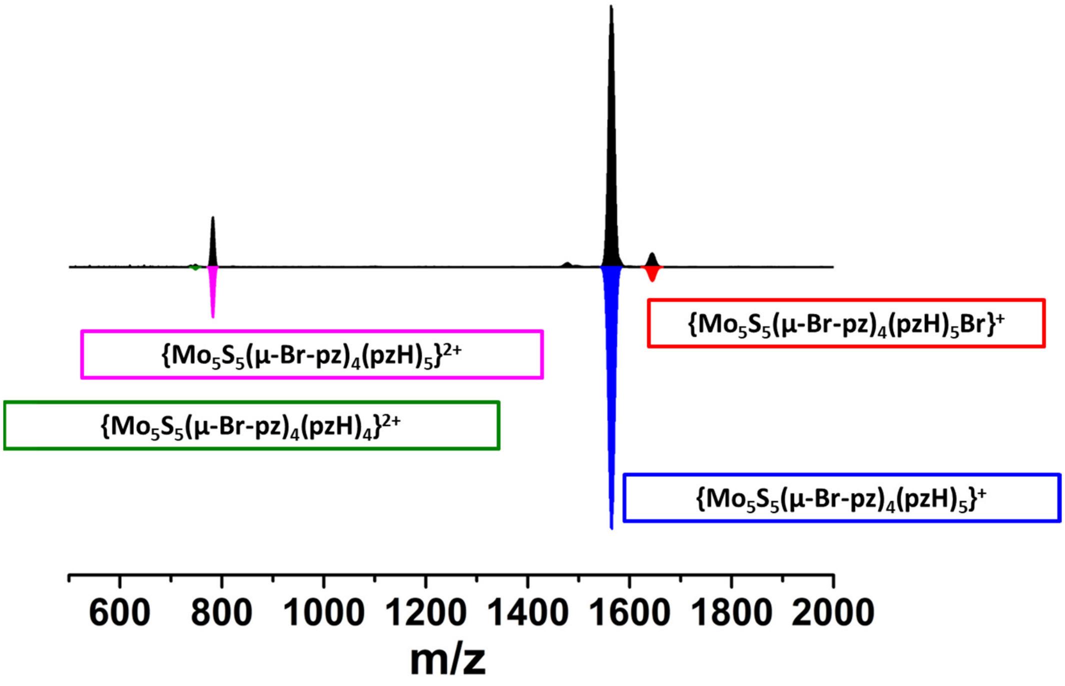

Figure S13. HR-ESI-MS spectrum of solution of **[3]Br$_2$** in dichloromethane (black) and simulations of cluster forms (colored).

NMR spectroscopy data for [4]Br

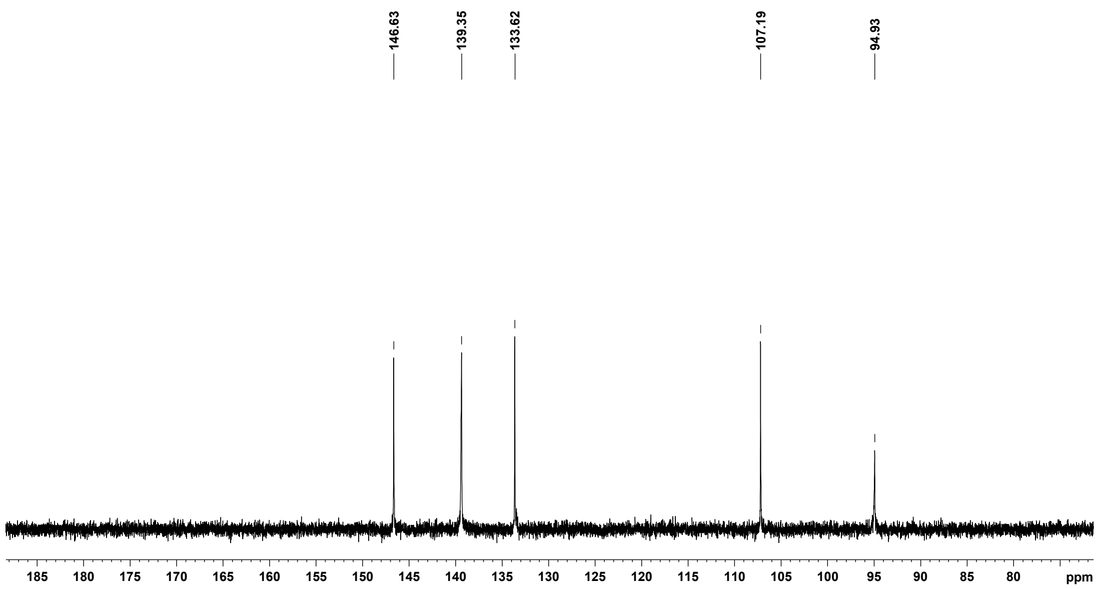

Figure S14. ^{13}C NMR spectrum of **[4]Br** in DMSO-d_6.

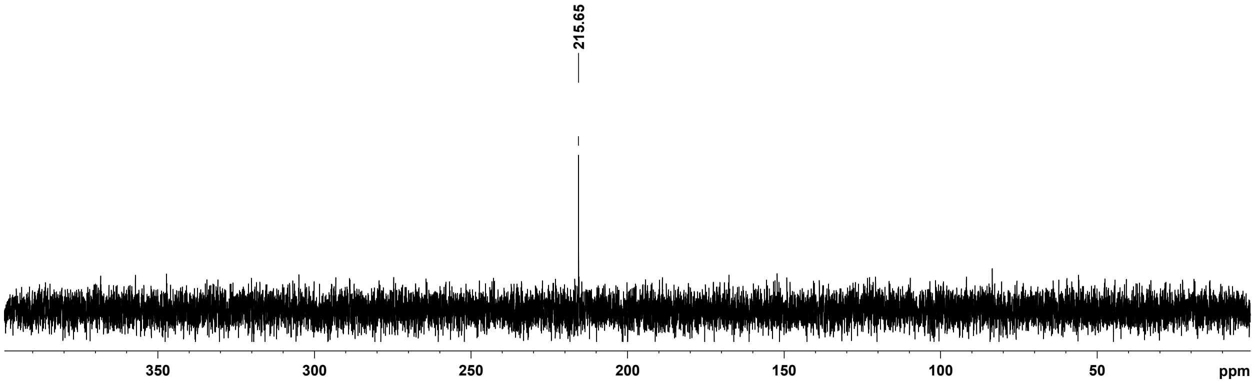

Figure S15. ^{15}N NMR spectrum of **[4]Br** in DMSO-d_6.

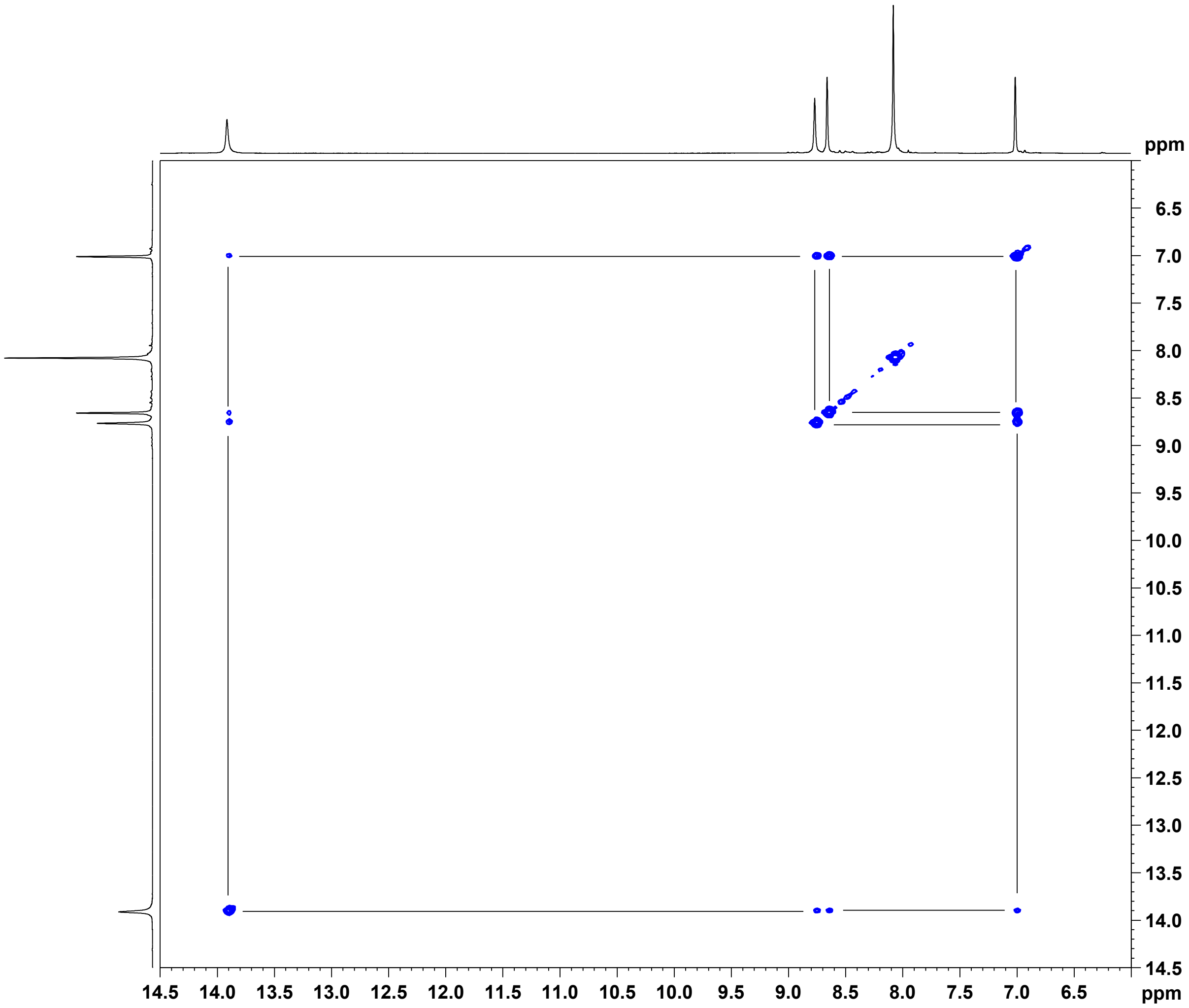

Figure S16. $^{1}H,^{1}H$-NMR correlation spectra of **[4]Br** in DMSO-d_6.

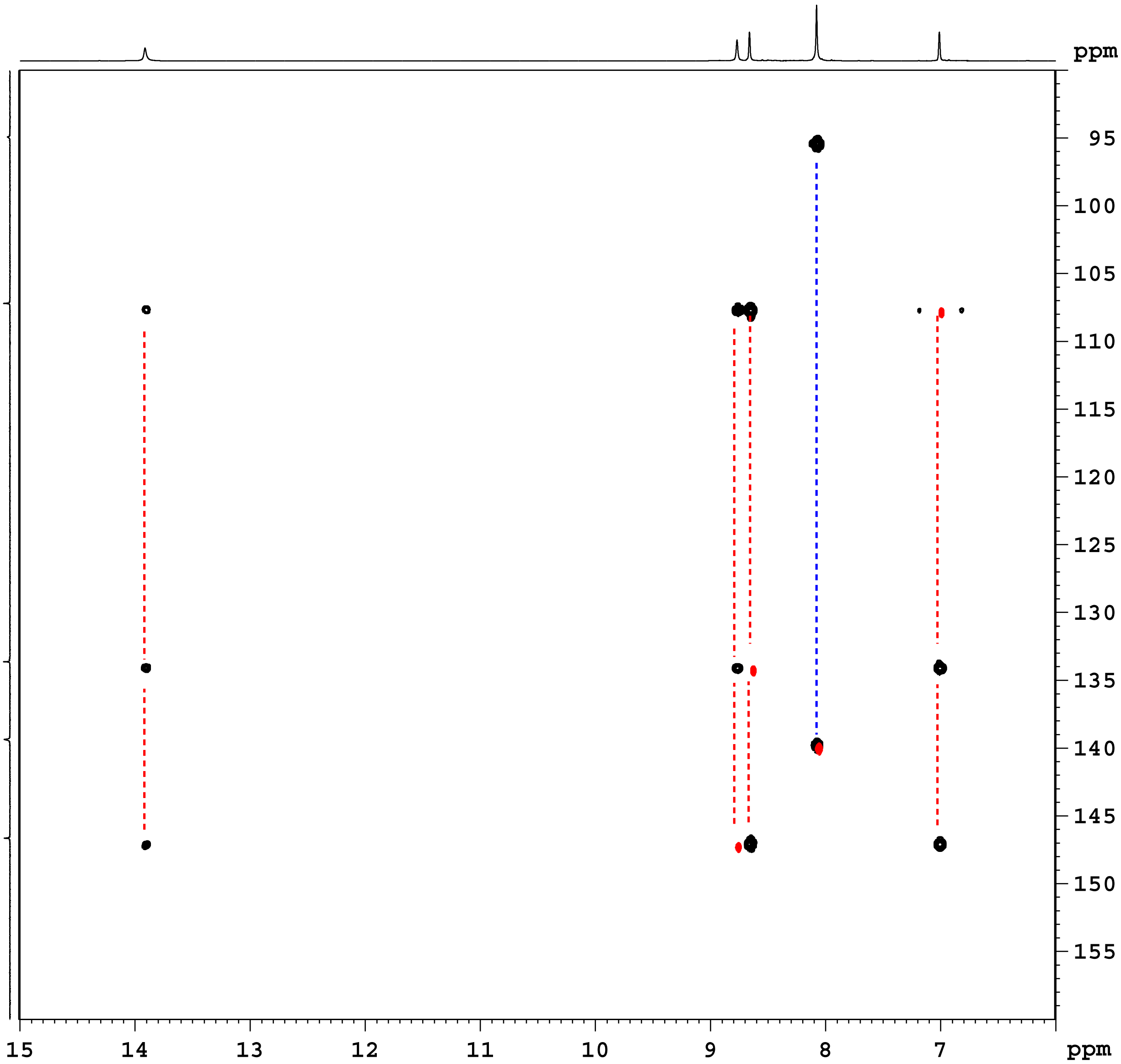

Figure S17. ¹H,¹³C-NMR correlation spectra (HMBC – black, HSQC – red) of **[4]Br** in DMSO-d_6.

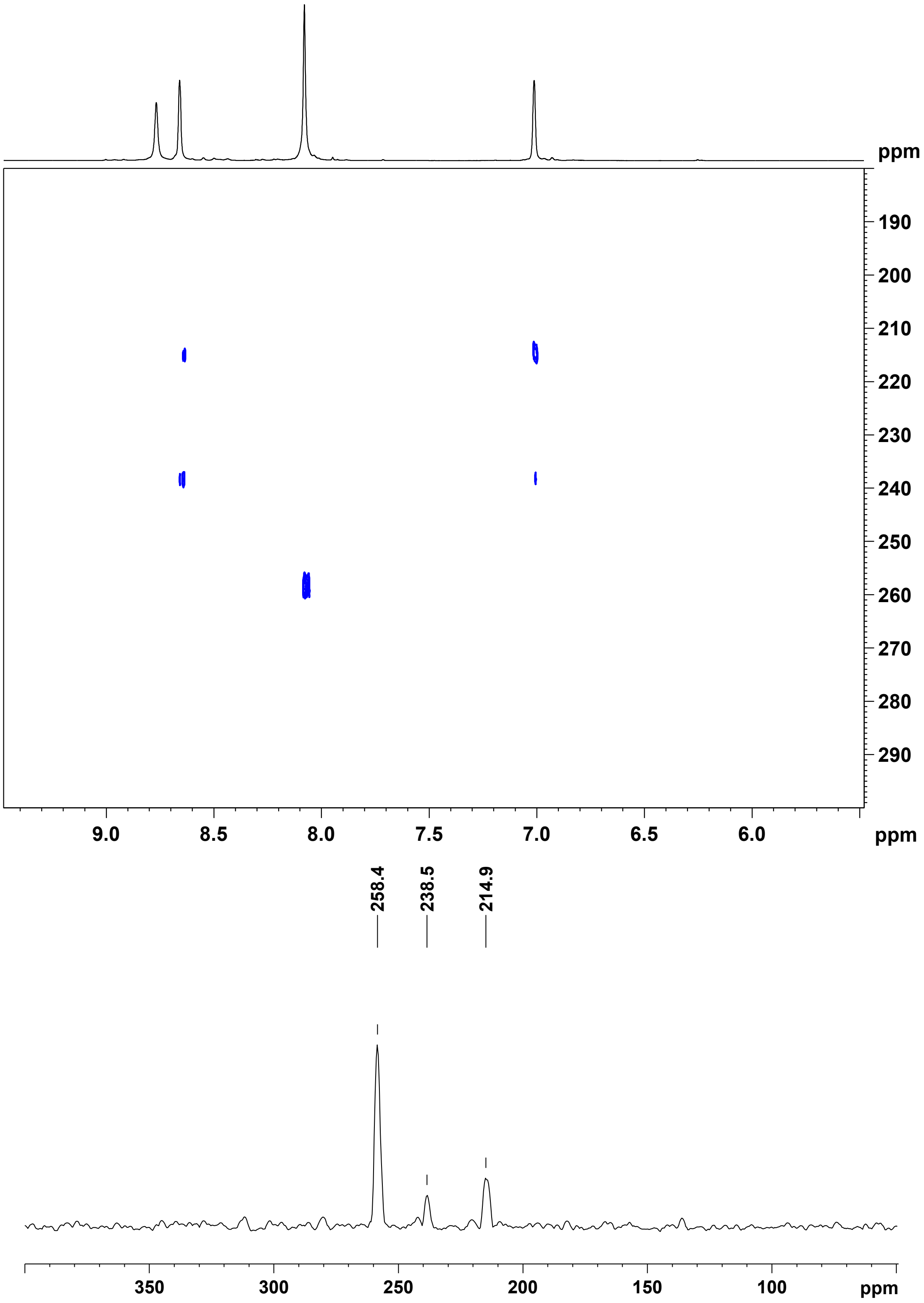

Figure S18. ^{1}H-^{15}N HMBC NMR spectra (top) and ^{15}N-axe projection (bottom) of **[4]Br** in DMSO-d_6.

Mass-spectrometry data for [4]Br

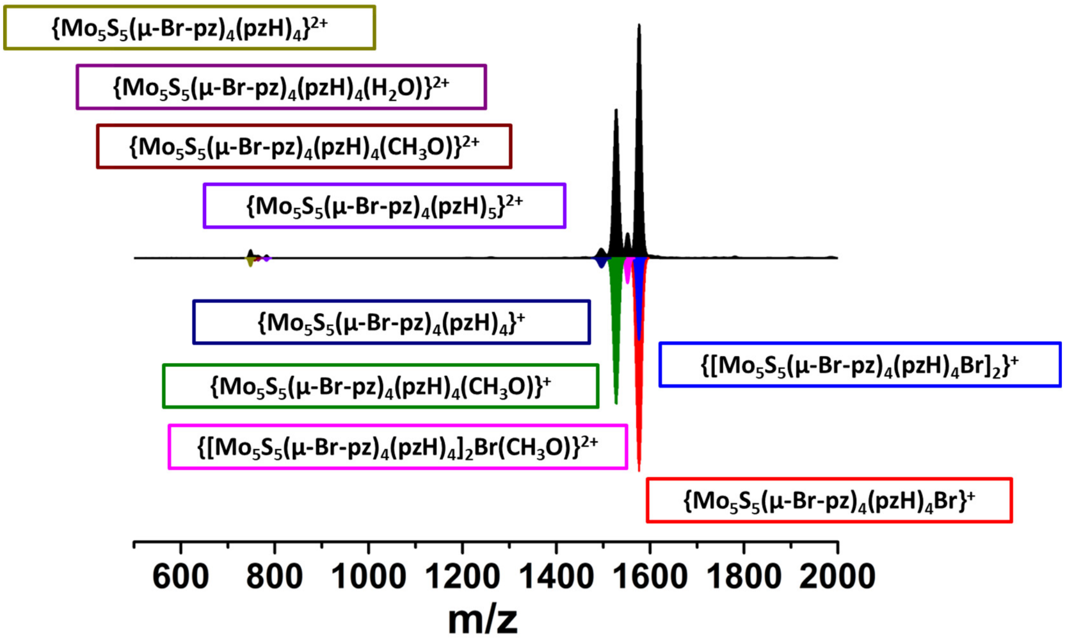

Figure S19. HR-ESI-MS spectrum of solution of **[4]Br** in acetonitrile (black) and simulations of cluster forms (colored).

Packing in the crystal structures

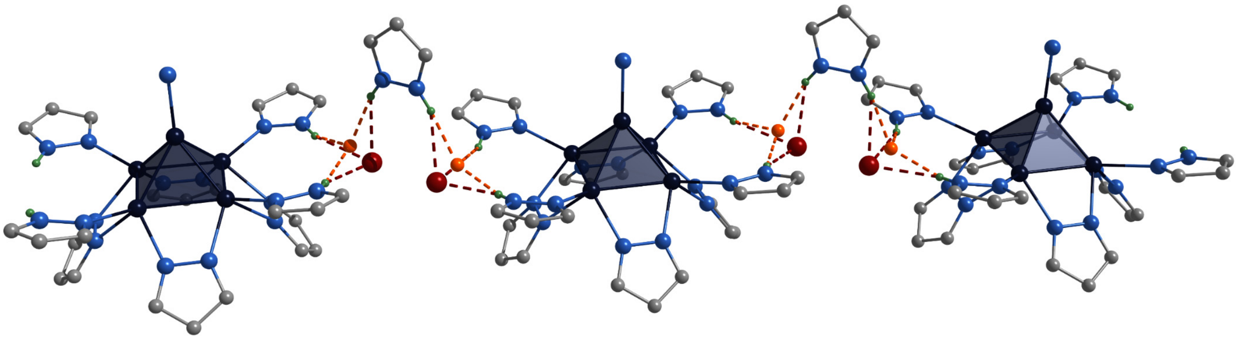

Figure S20. Packing in the crystal structure of **[1^{red}]Br**: hydrogen bonds between terminal pyrazole ligands, Br/O and solvate pyrazole molecules forming infinite chains. Color code: Mo – dark blue, C – gray, N – blue, Br – red, O – orange, H – green, N-H···Br – red, N-H···O – orange. Hydrogen and sulfur atoms are omitted for clarity.

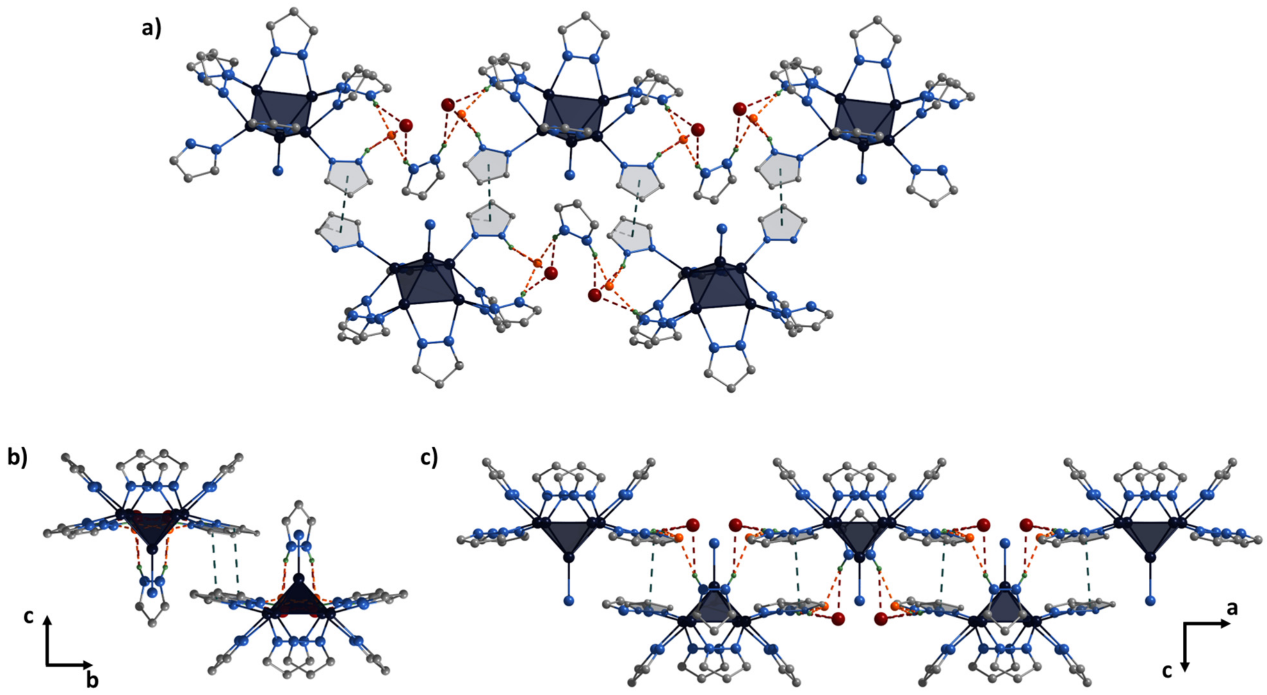

Figure S21. Packing in the crystal structure of **[1^{red}]Br**. a) Side view on the packing of the chains by π-π stacking interactions forming infinite layers. b) View on the layer along *a* axis. c) View on the layer along *b* axis. Color code: Mo – dark blue, C – gray, N – blue, Br – red, O – orange, H – green, N-H···Br – red, N-H···O – orange, π-π stacking – green. Hydrogen and sulfur atoms are omitted for clarity.

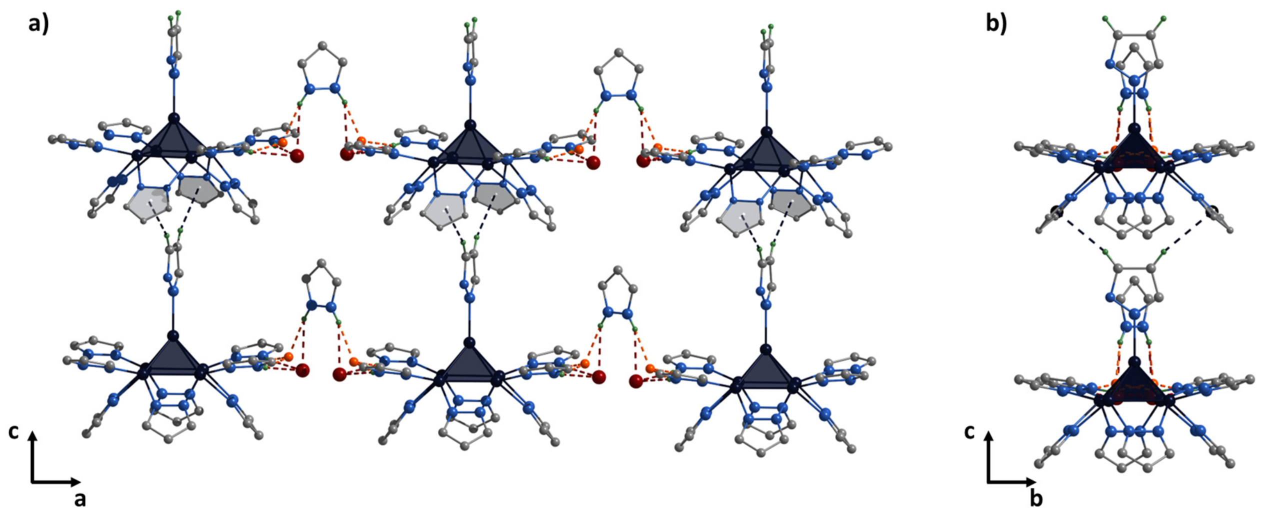

Figure S22. Packing in the crystal structure of **[1^{red}]Br**. a) Packing of the layers by C-H-π stacking interactions: view along *b* axis (a) and *a* axis (b). Color code: Mo – dark blue, C – gray, N – blue, Br – red, O – orange, H – green, N-H···Br – red, N-H···O – orange, C-H-π stacking – black. Hydrogen and sulfur atoms are omitted for clarity.

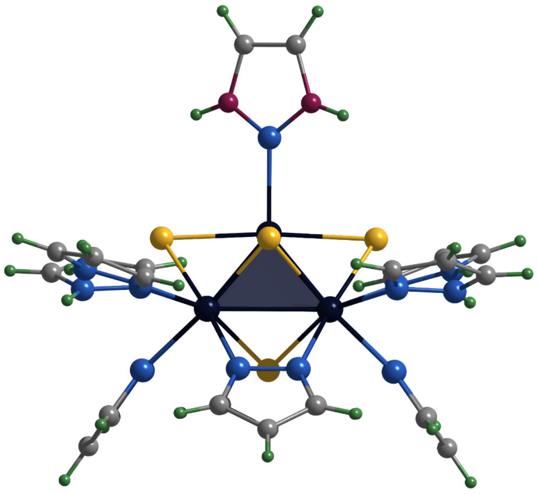

Figure S23. Crystal structure of cationic cluster $[\{Mo_5(\mu_3\text{-}S)^i{}_4(\mu_4\text{-}S)^i(\mu\text{-}pz)^i{}_4\}(pzH)^t{}_5]^{2+}$, side view. Color code: Mo_5 – dark blue square pyramid, S – yellow, N – blue, C – grey, H – green, disordered C/N – purple.

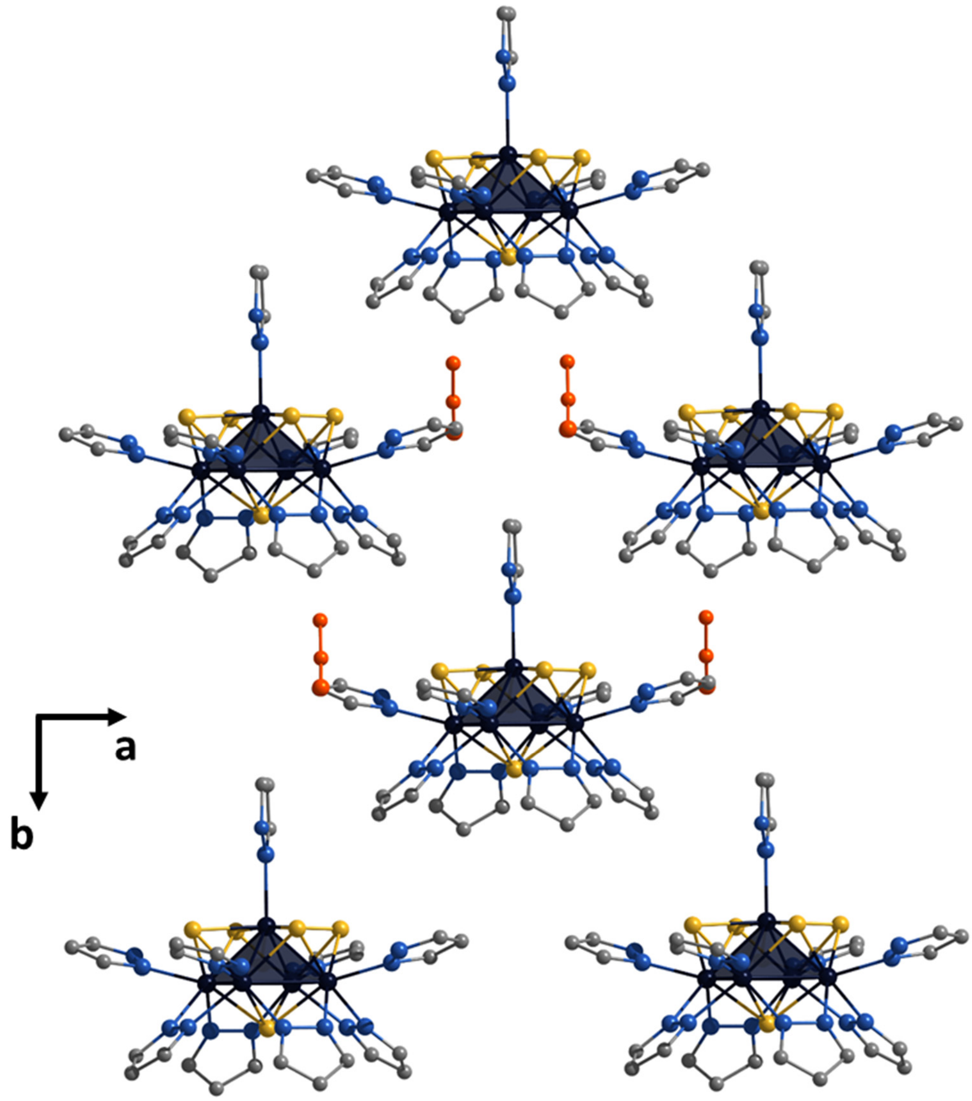

Figure S24. Packing of the clusters and solvate acetonitrile molecules along *ab* plane in the crystal structure of **[1^{ox}][Mo_6I_{14}]** forming infinite layers. Color code: Mo – dark blue, C – gray, N – blue, S – yellow, CH_3CN – orange. Hydrogen atoms are omitted for clarity.

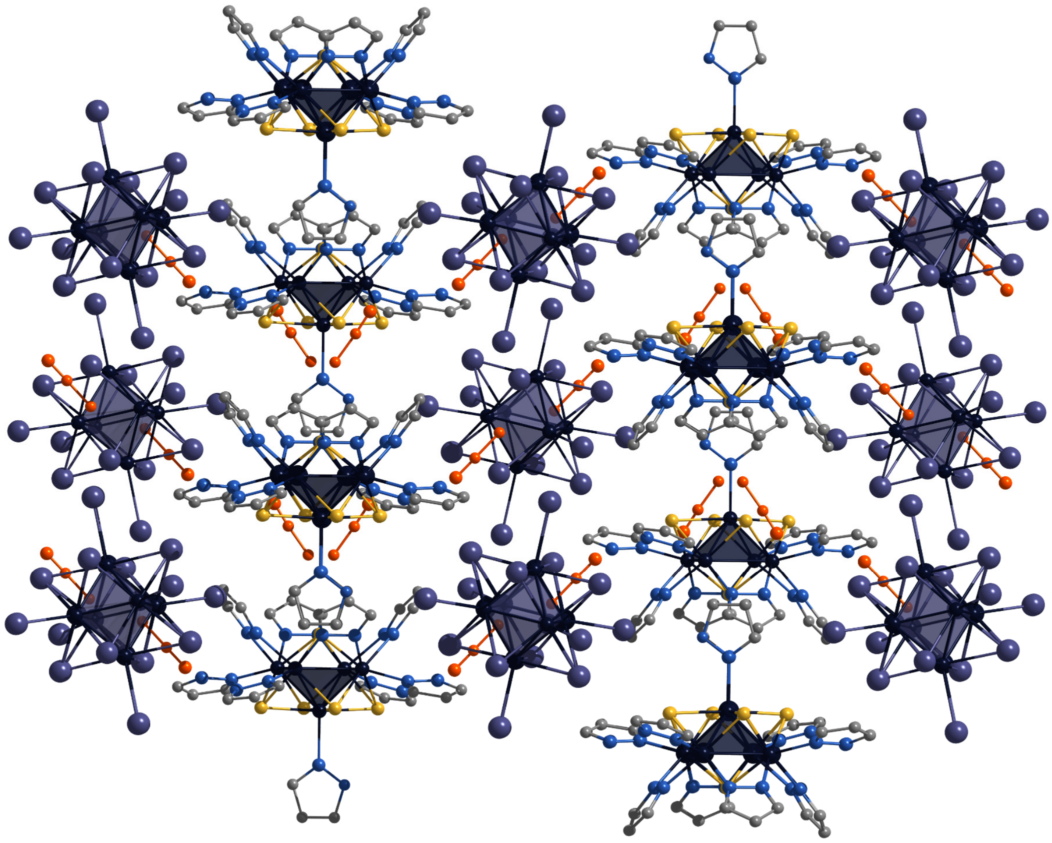

Figure S25. Packing of the layers in the crystal structure of **[1^{ox}][Mo$_6$I$_{14}$]**. Color code: Mo – dark blue, C – gray, N – blue, S – yellow, I – purple, CH_3CN – orange. Hydrogen atoms are omitted for clarity.

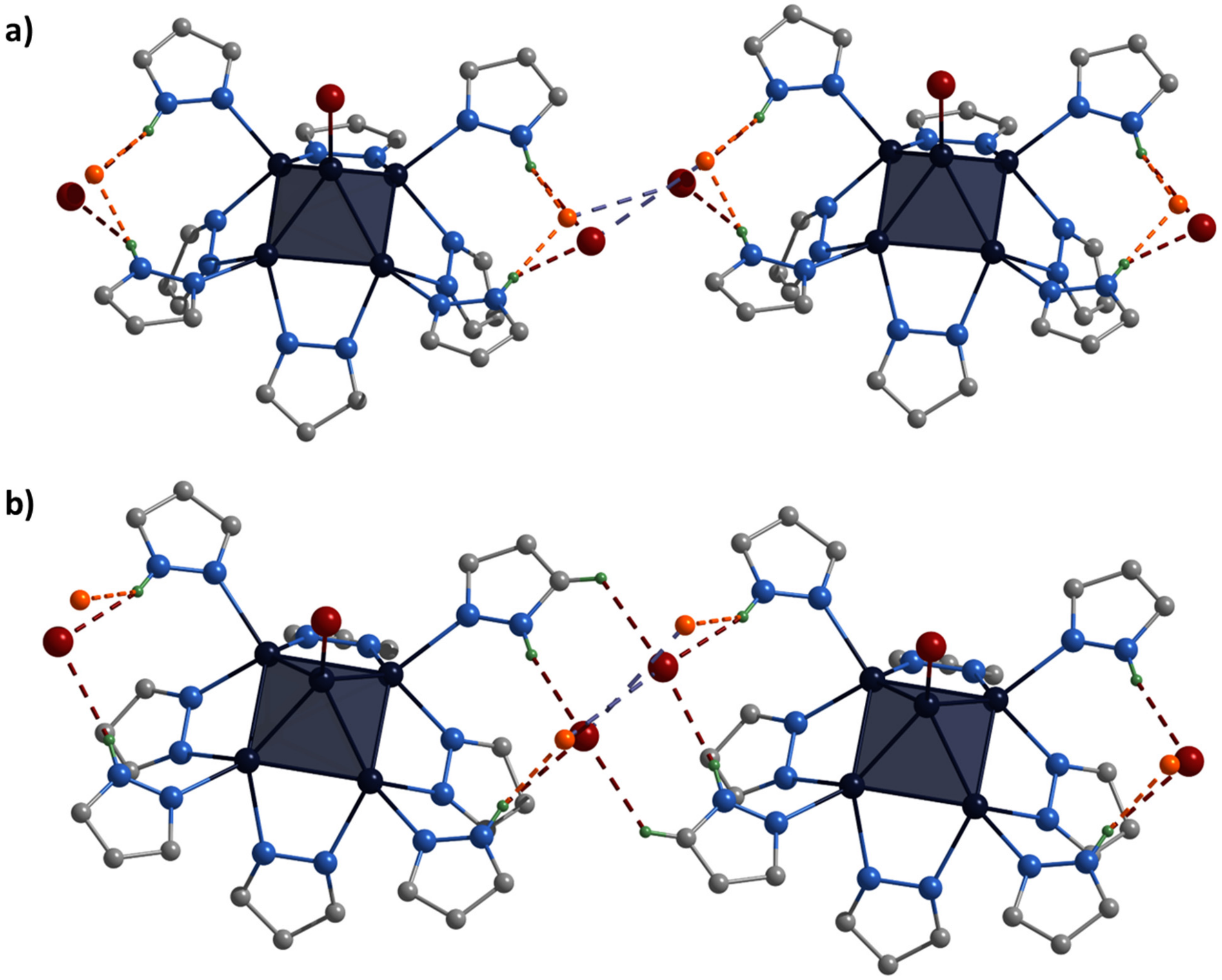

Figure S26. Packing in the crystal structure of **[2]Br**·2.5H_2O·0.5DMF. Chains formed by hydrogen bonds between basal pyrazole ligands and Br-anions or solvate water molecules along *a* (a) or *c* axis (b). Color code: Mo – dark blue, C – gray, N – blue, Br – red, O – orange, H – green, N-H···Br – red, N-H···O – orange, Br···O – violet. Hydrogen and sulfur atoms are omitted for clarity.

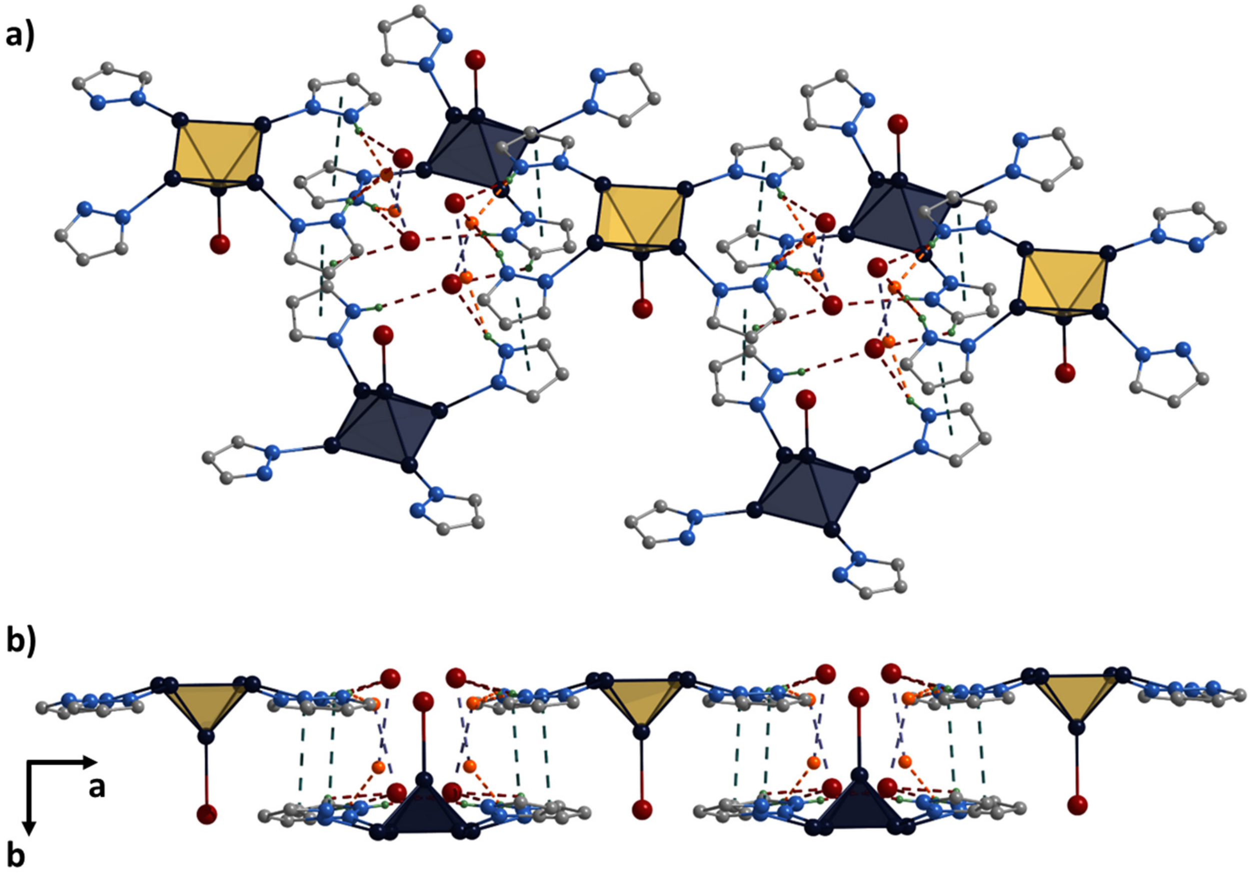

Figure S27. Packing in the crystal structure of **[2]Br**·2.5H_2O·0.5DMF. Packing of the chains by π-π stacking interactions between basal pyrazole ligands: side view *a* (a) and view along *c* axis (b). Color code: Mo – dark blue, C – gray, N – blue, Br – red, O – orange, H – green, N-H···Br – red, N-H···O – orange, Br···O – violet, π-π stacking – green. Different colors of the square pyramids indicate that the clusters belong to different chains. Hydrogen and sulfur atoms are omitted for clarity.

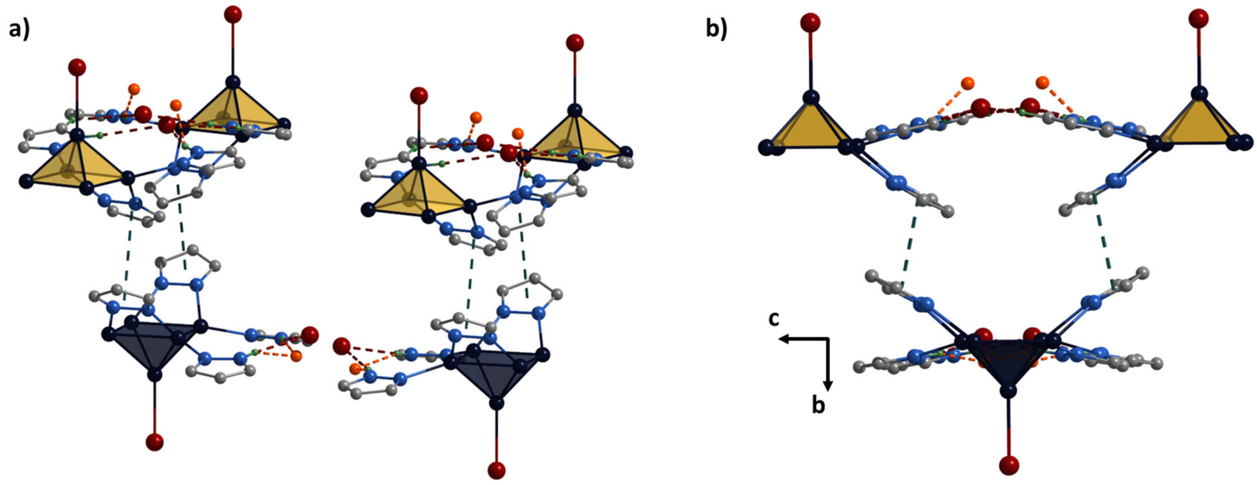

Figure S28. Packing in the crystal structure of **[2]Br**·2.5H_2O·0.5DMF. Packing of the chains by π-π stacking interactions between pyrazolate ligands: side view *a* (a) and view along *a* axis (b). Color code: Mo – dark blue, C – gray, N – blue, Br – red, O – orange, H – green, N-H···Br – red, N-H···O – orange, Br···O – violet, π-π stacking – green. Different colors of the square pyramids indicate that the clusters belong to different chains. Hydrogen and sulfur atoms are omitted for clarity.

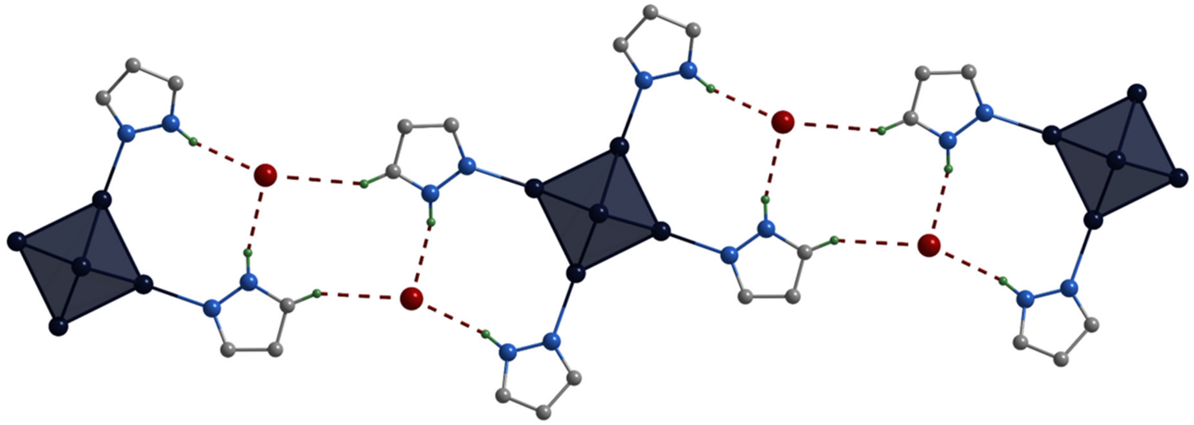

Figure S29. Packing in the crystal structure of **[3]Br$_2$**·CH_3CN·Et_2O. Hydrogen bonds formed between basal pyrazole ligands and Br-anions forming infinite chains. Color code: Mo – dark blue, C – gray, N – blue, Br – red, H – green, N-H···Br / C-H···Br – red. Hydrogen and sulfur atoms are omitted for clarity.

a)

b)

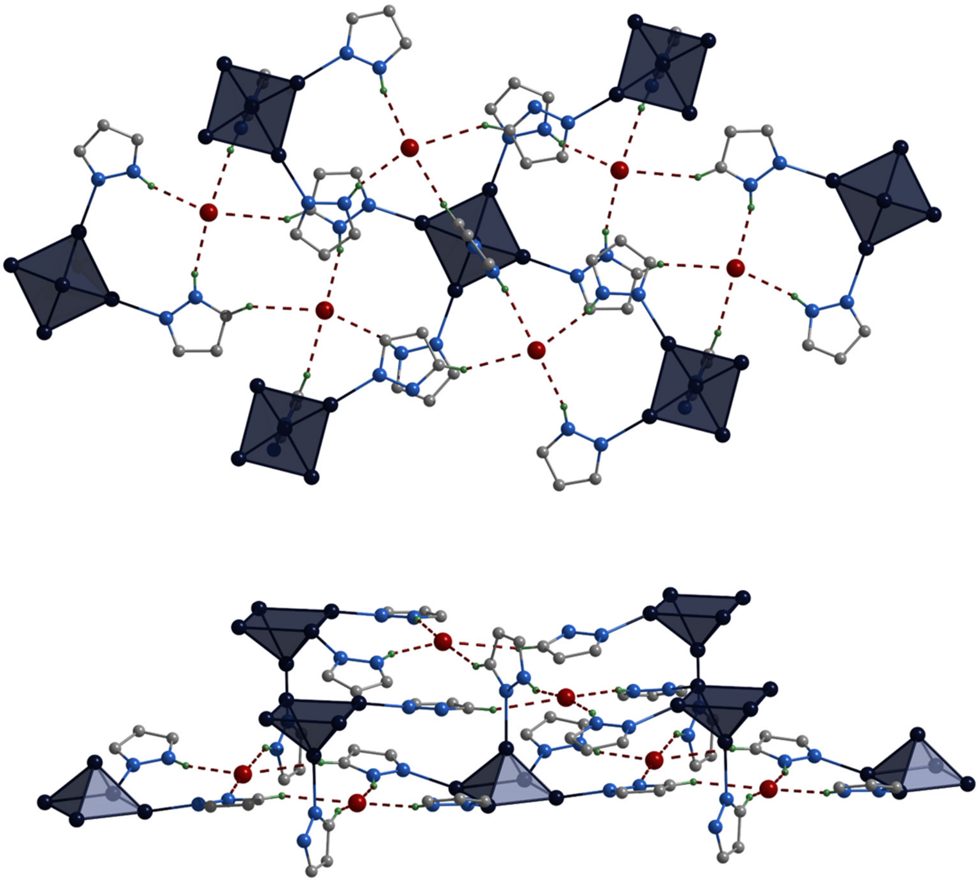

Figure S30. Packing in the crystal structure of **[3]Br₂**·CH_3CN·Et_2O. Connection of chains by hydrogen bonds between apical pyrazole ligands and Br-anions: top (a) and side (b) view. Color code: Mo – dark blue, C – gray, N – blue, Br – red, H – green, N-H···Br / C-H···Br – red. Hydrogen and sulfur atoms are omitted for clarity.

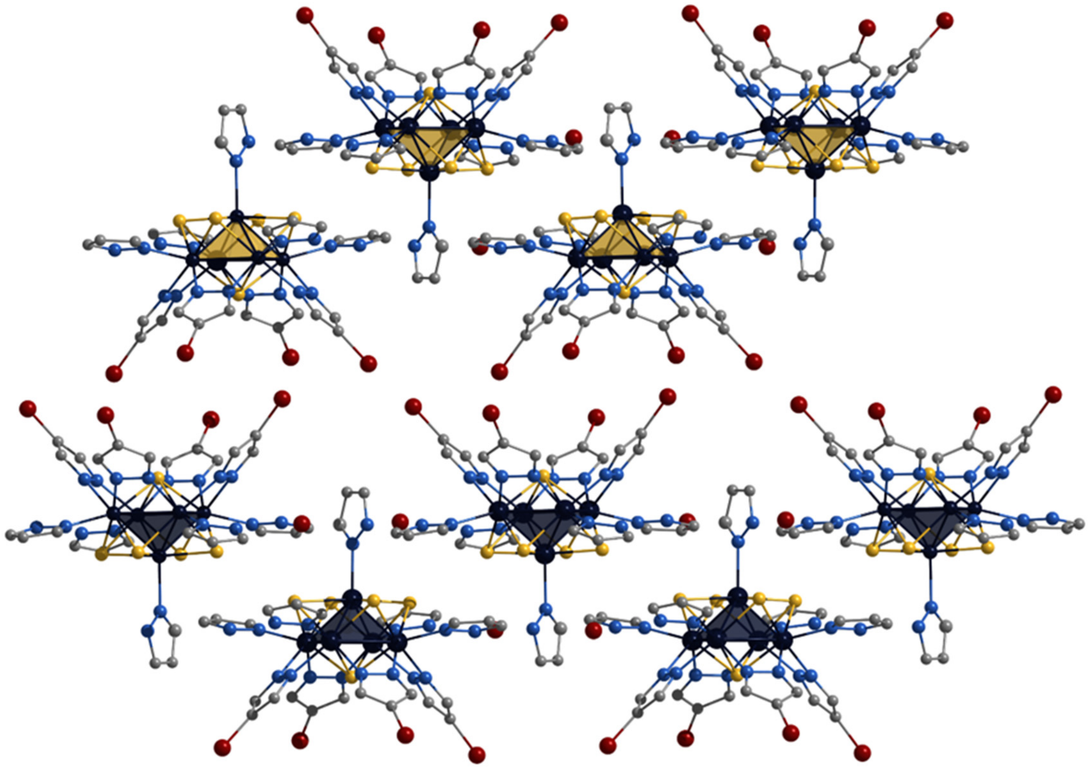

Figure S31. Packing of layers in the crystal structure of **[3]Br$_2$**·CH_3CN·Et_2O. Color code: Mo – dark blue, C – gray, S – yellow, N – blue, Br – red. Different colors of the square pyramids indicate that the clusters belong to different layers. Hydrogen atoms are omitted for clarity.

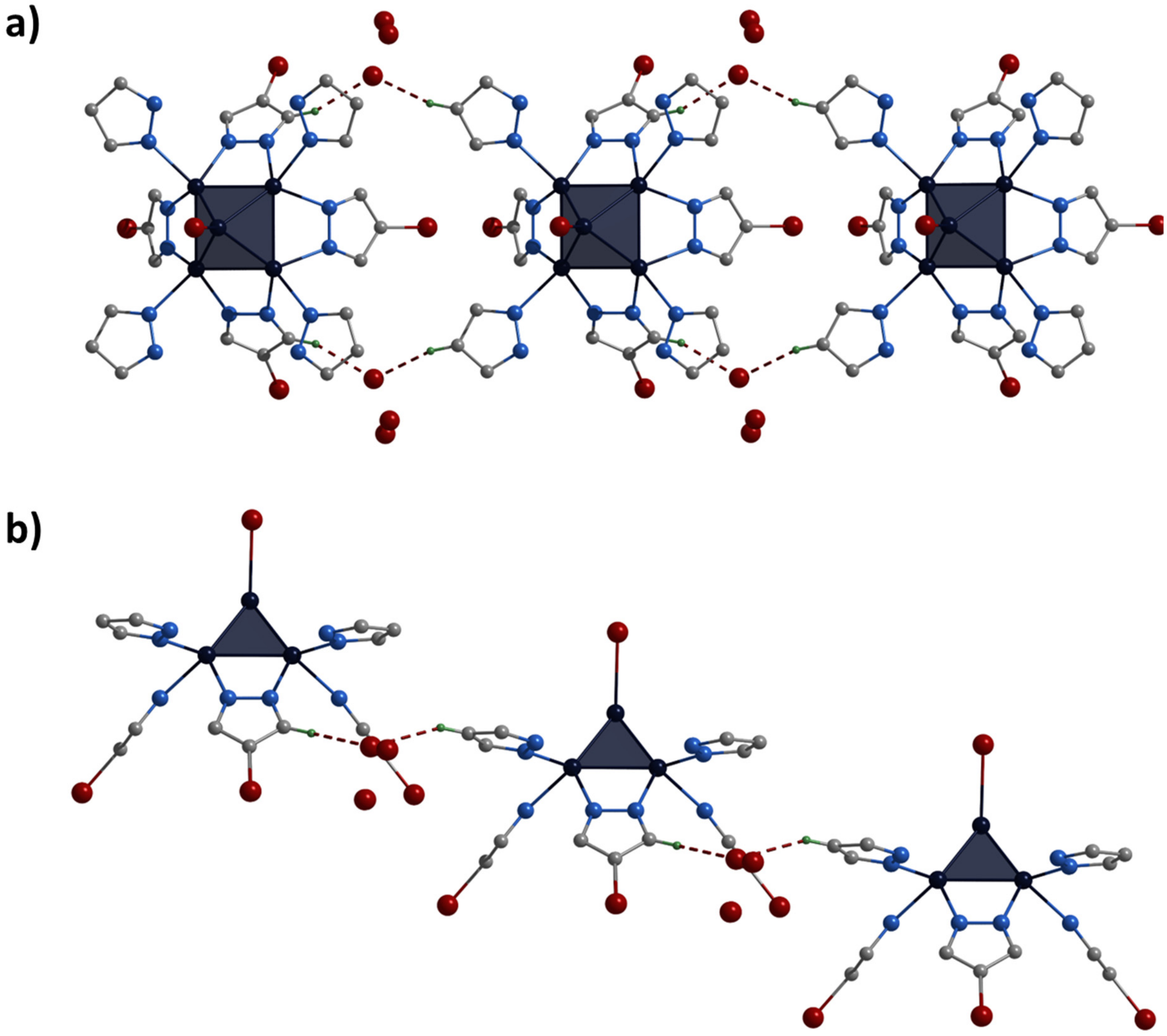

Figure S32. Packing in the crystal structure of **[4]Br**·1.25H_2O·2DMSO. Chains formed by hydrogen bonds between basal pyrazole ligands, Br-anions and pyrazolate ligands: top (a) and side (b) view. Color code: Mo – dark blue, C – gray, N – blue, Br – red, C-H···Br – red. Hydrogen and sulfur atoms are omitted for clarity.

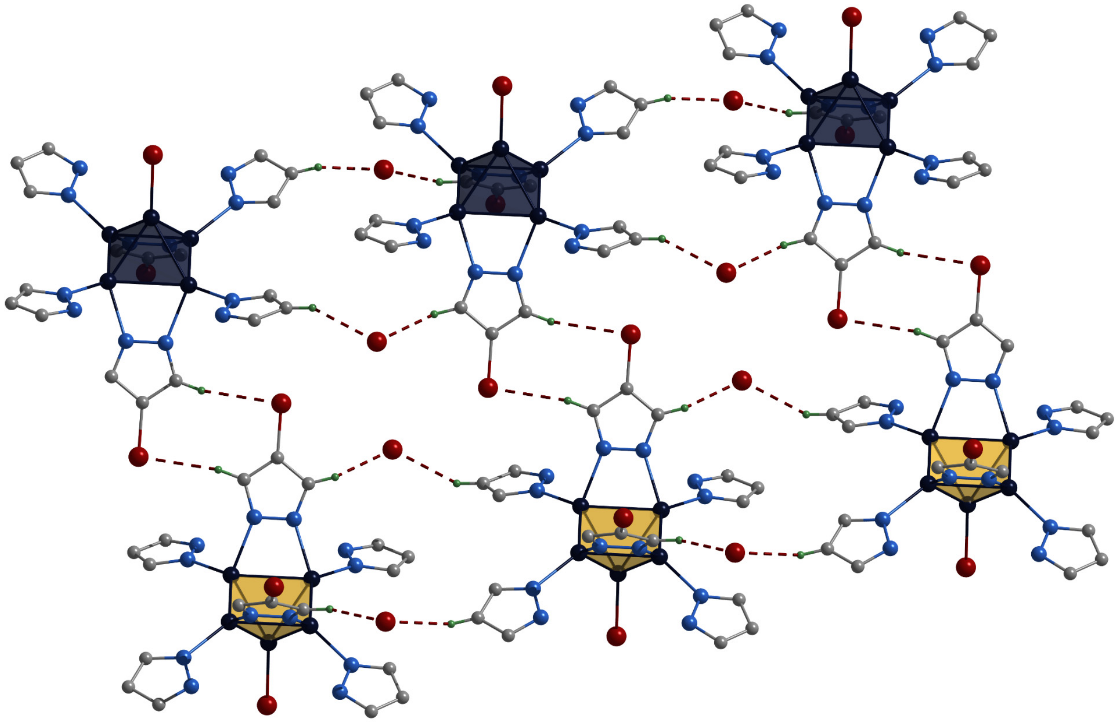

Figure S33. Packing in the crystal structure of **[4]Br**·1.25H_2O·2DMSO. Connection of the chains by hydrogen bonds between pyrazolate ligands. Color code: Mo – dark blue, C – gray, N – blue, Br – red, C-H···Br – red. Different colors of the square pyramids indicate that the clusters belong to different chains. Hydrogen and sulfur atoms are omitted for clarity.

EPR and DFT calculations data for [1^{red}]Br

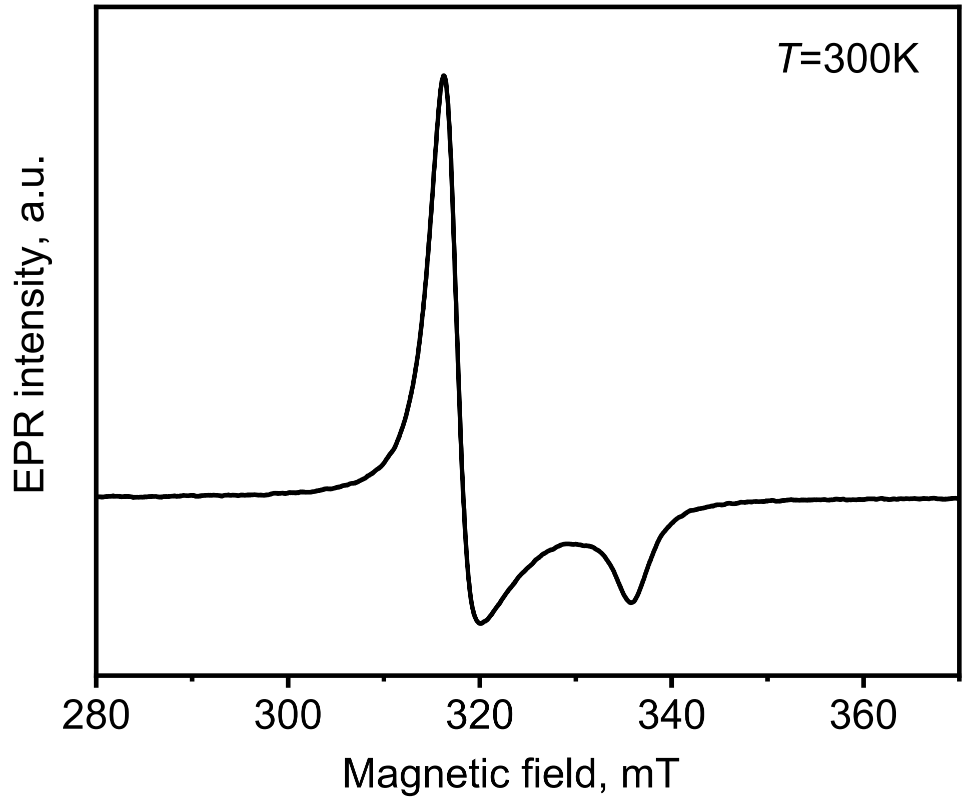

Figure S34. EPR spectrum measured at 300 K of **[1^{red}]Br**.

Table S1. The EPR parameters calculated for [{$Mo_5(\mu_3\text{-}S)_4(\mu_4\text{-}S)(\mu\text{-}pz)_4$}$(pzH)_5$]$^+$ cation at the different levels of theory.

	ZORA-SCALAR-UB3LYP/TZP	ZORA-SO-UB3LYP/TZP	Experimental
**[1^{red}]Br**	g_{xx} = 2.14,<br>g_{yy} = 2.12,<br>g_{zz} = 1.98	g_{xx} = 2.14,<br>g_{yy} = 2.13,<br>g_{zz} = 1.98	g_{xx} = 2.12,<br>g_{yy} = 2.11,<br>g_{zz} = 1.99

Cyclic voltammetry data

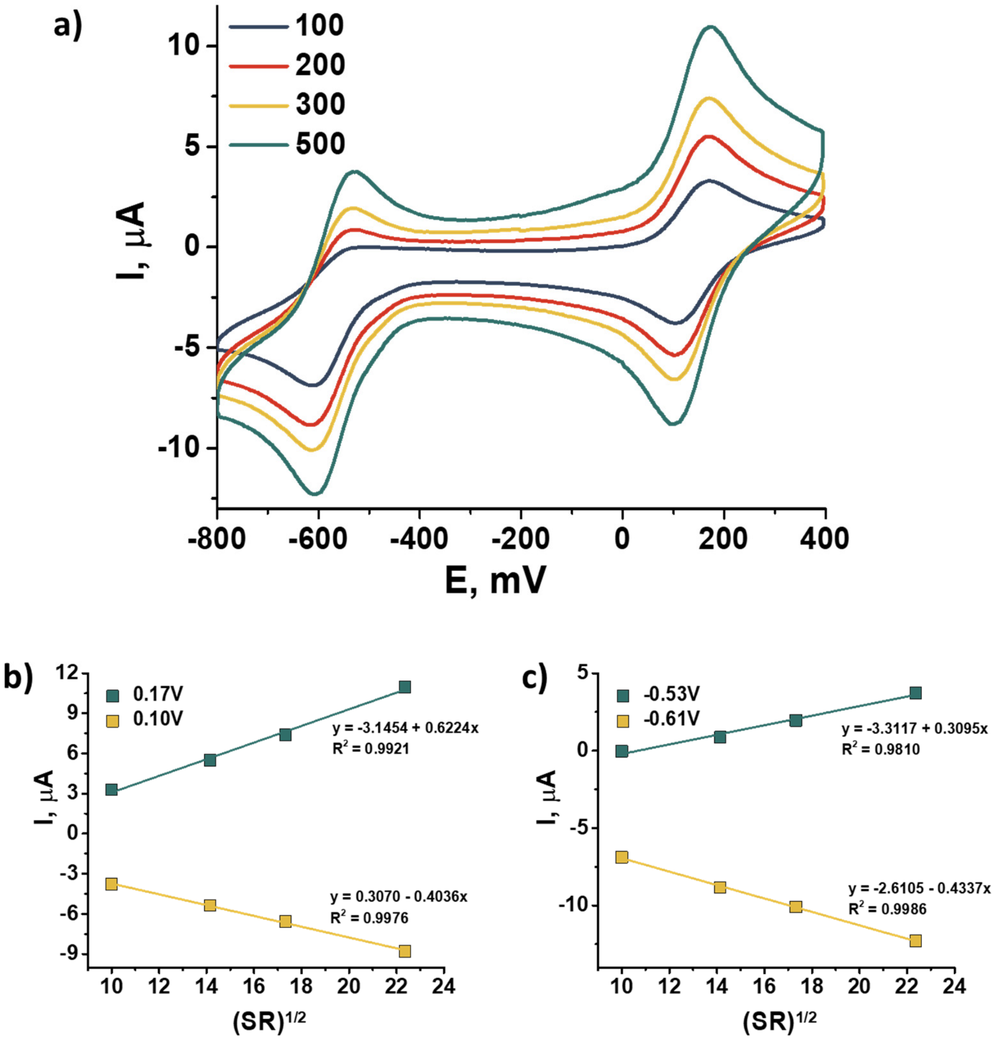

Figure S35. Cyclic voltammetry of the $\mathbf{[1^{ox}]Br_2}$ (0.5 mM) in 0.1 M Bu_4NClO_4 acetonitrile solution at different scan rates (a). Linear dependence of the anodic and cathodic (b, c) peak current upon square root of the potential scan rate $\sqrt{SR}$;

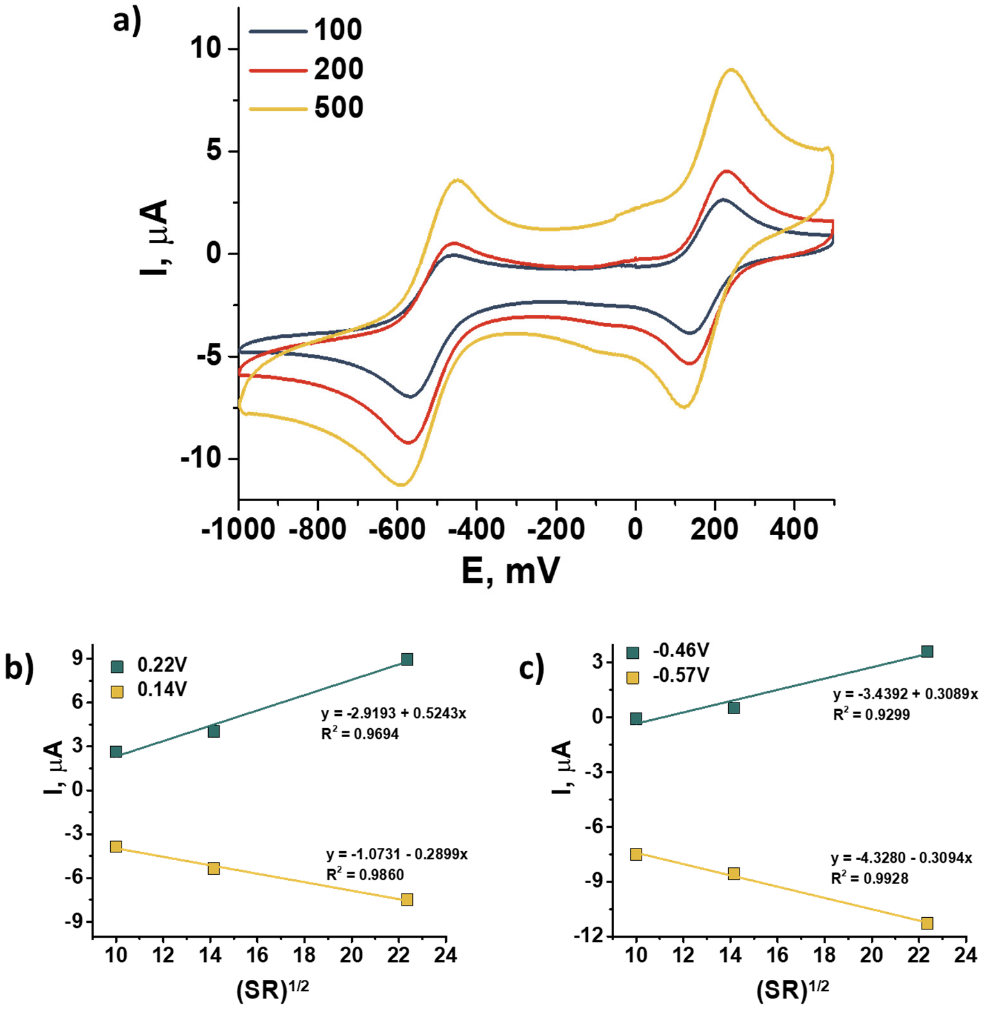

Figure S36. Cyclic voltammetry of the **[1^{ox}]Br$_2$** (0.5 mM) in 0.1 M Bu_4NClO_4 dichloromethane solution at different scan rates (a). Linear dependence of the anodic and cathodic (b, c) peak current upon square root of the potential scan rate $\sqrt{SR}$;

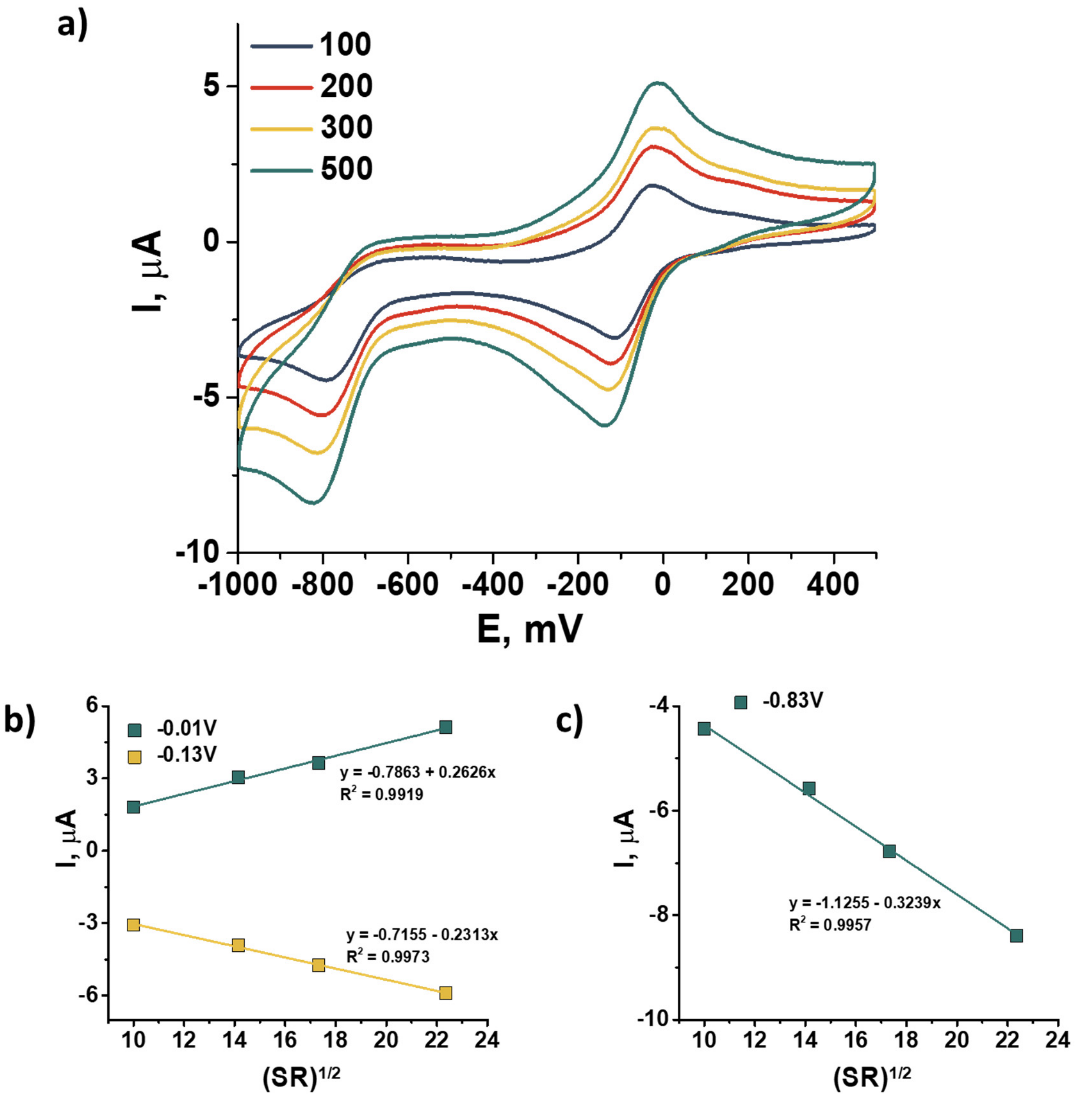

Figure S37. Cyclic voltammetry of the **[2]Br** (0.5 mM) in 0.1 M Bu_4NClO_4 dichloromethane solution at different scan rates (a). Linear dependence of the anodic and cathodic (b, c) peak current upon square root of the potential scan rate $\sqrt{SR}$;

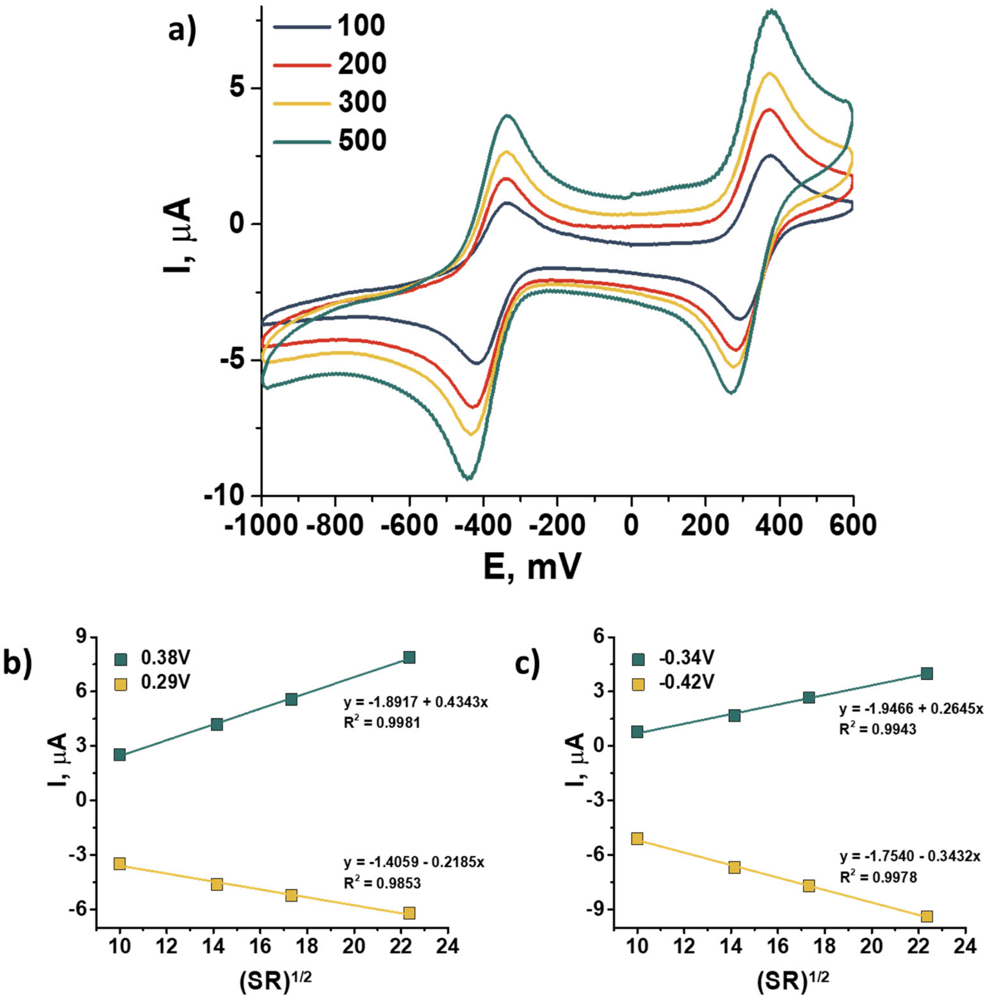

Figure S38. Cyclic voltammetry of the **[3]Br$_2$** (0.5 mM) in 0.1 M Bu_4NClO_4 dichloromethane solution at different scan rates (a). Linear dependence of the anodic and cathodic (b, c) peak current upon square root of the potential scan rate $\sqrt{SR}$;

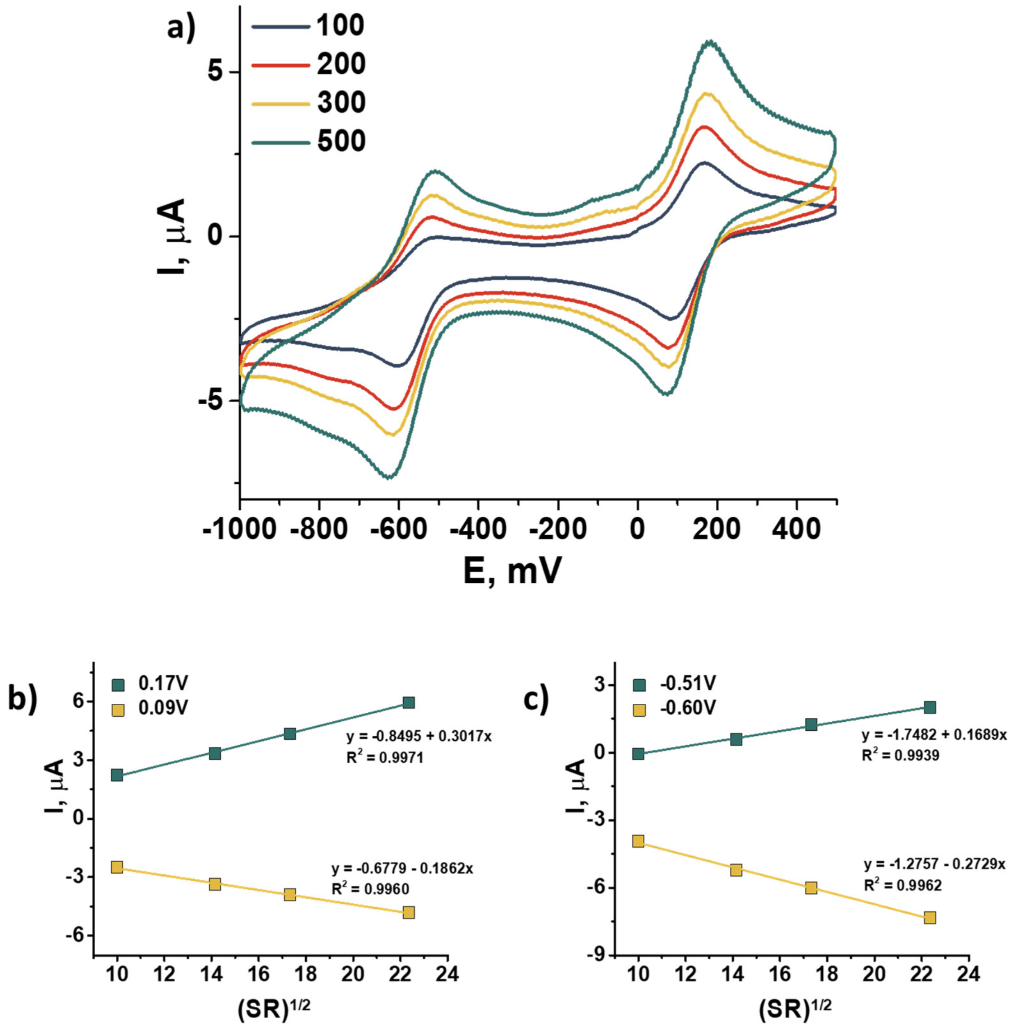

Figure S39. Cyclic voltammetry of the **[4]Br** (0.5 mM) in 0.1 M Bu_4NClO_4 dichloromethane solution at different scan rates (a). Linear dependence of the anodic and cathodic (b, c) peak current upon square root of the potential scan rate $\sqrt{SR}$;

DFT calculations data

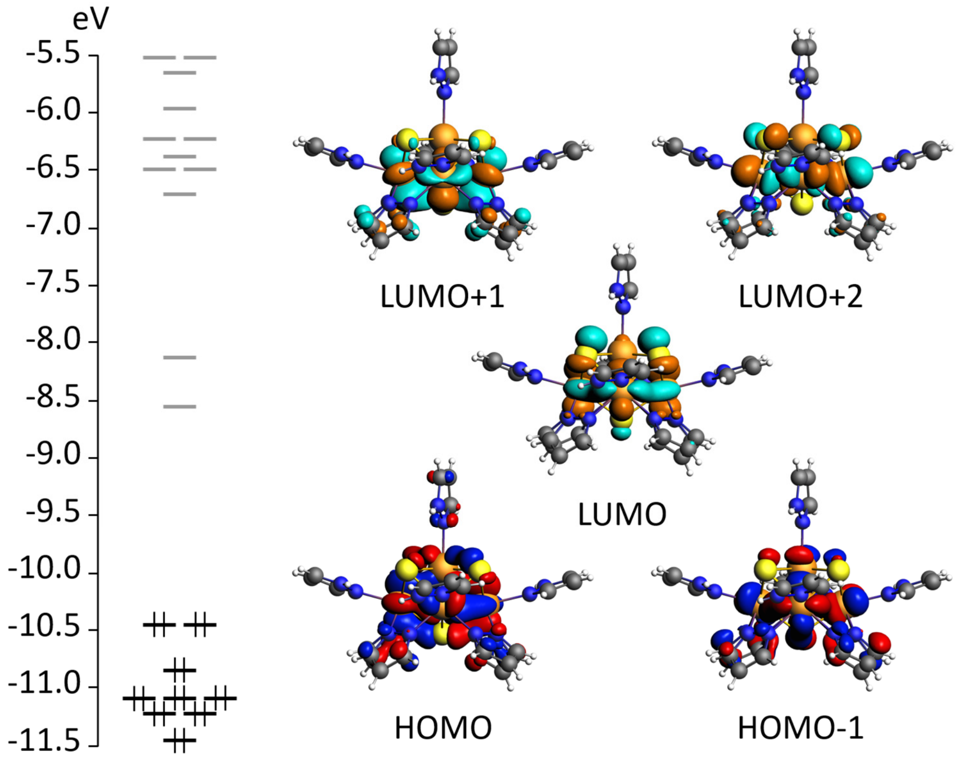

Figure S40. Energy levels diagram and frontier molecular orbitals of the $[\{Mo_5(\mu_3\text{-}S)_4(\mu_4\text{-}S)(\mu\text{-}pz)_4\}(pzH)_5]^{2+}$ cluster.

Table S2. Orbital energies, symmetry of molecular orbitals and percentage contributions from the metal atoms, sulfur ligands and pzH molecules for the $[\{Mo_5(\mu_4\text{-}S)(\mu_3\text{-}S)_4(\mu\text{-}pz)_4\}(pzH)_5]^{2+}$ cluster.

MO	Energy (eV)	Mo_5	μ_4-S	μ_3-S	μ-pz	Outer pz
307 (LUMO+2)	-6.713	80.54	-	19.46	-	-
306 (LUMO+1)	-8.132	78.95	1.76	7.51	11.78	-
305 (LUMO)	-8.559	72.37	2.58	25.04	-	-
304 (HOMO)	-10.454	78.47	5.41	8.16	6.48	1.48
303 (HOMO-1)	-10.517	84.10	5.43	3.04	7.44	-
302 (HOMO-2)	-10.845	82.62	-	12.51	-	4.87

UV-vis-NIR absorption data

Table S3. Main absorption bands (λ, nm) and corresponding extinction coefficients (ε, $L \cdot mol^{-1} \cdot mm^{-1}$) in UV-vis-NIR absorption spectra.

Compound	Solvent	1st band<br>λ / ε	2nd band<br>λ / ε	3rd band<br>λ / ε
**[1^{red}]Br**	Acetonitrile	402 / 555	660 / 260	–
**[1^{ox}]Br_2**	Acetonitrile	420 / 635	640 / 195	798 / 295
**[2]Br**	Acetonitrile	434 / 550	652 / 230	878 / 180
**[3]Br_2**	Acetonitrile	432 / 650	606 / 290	808 / 305
**[4]Br**	DMSO	444 / 620	664 / 310	888 / 200

IR spectra

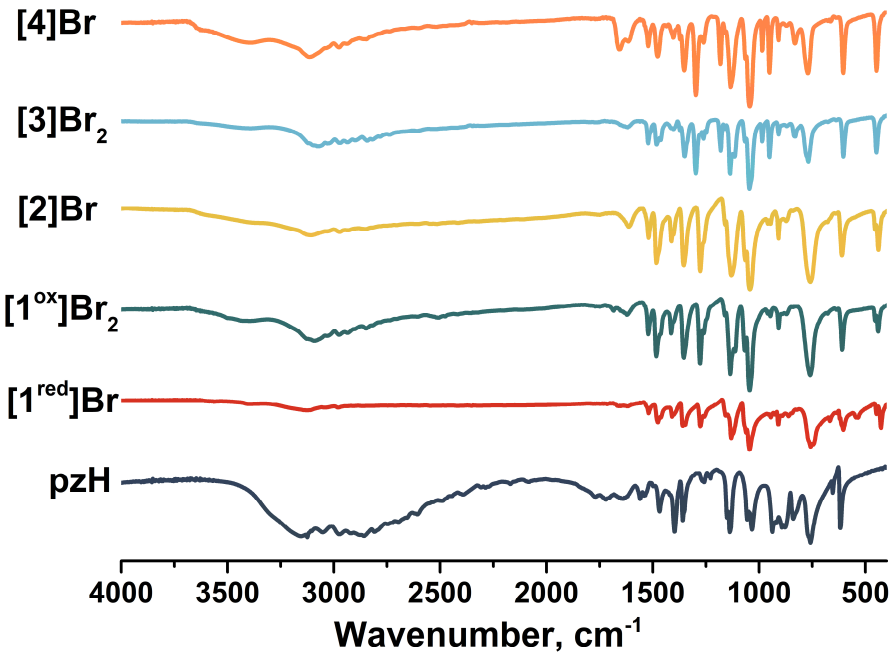

Figure S41. FTIR spectra of cluster obtained in comparison with pyrazole.

Thermogravimetric analysis

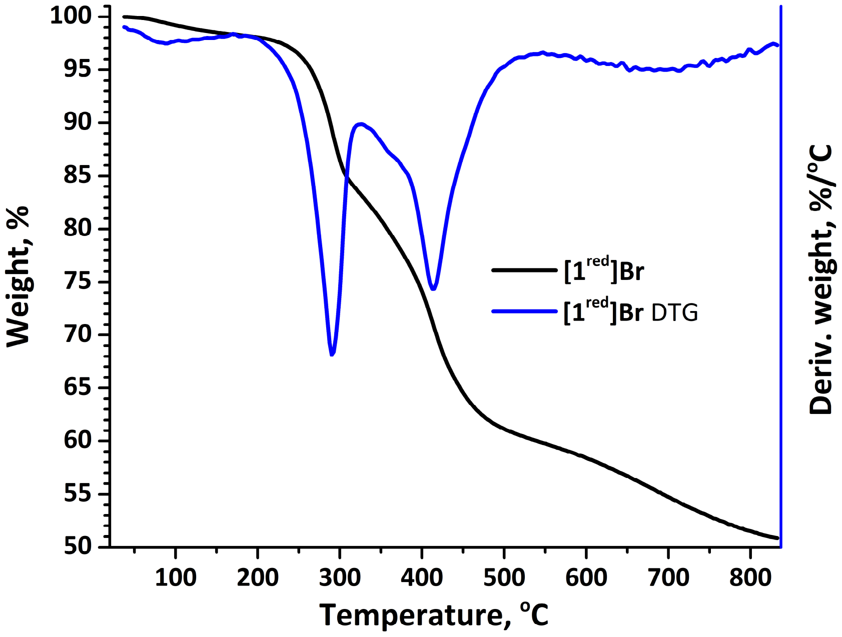

Figure S42. TGA and DTG curves of **[1^{red}]Br**. Heating rates of 10 ^{o}C·min^{-1}.

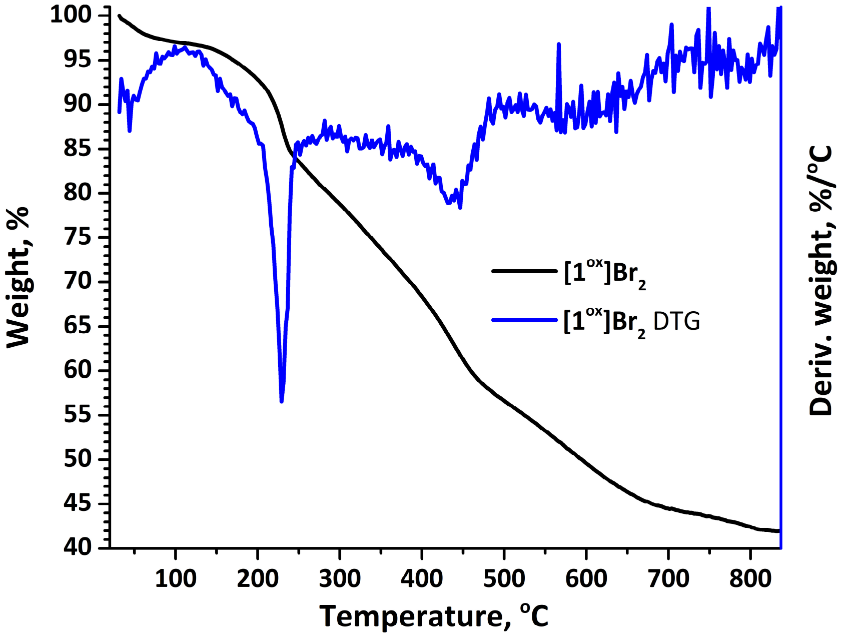

Figure S43. TGA and DTG curves of **[1^{ox}]Br$_2$**. Heating rates of 10 ^{o}C·min^{-1}.

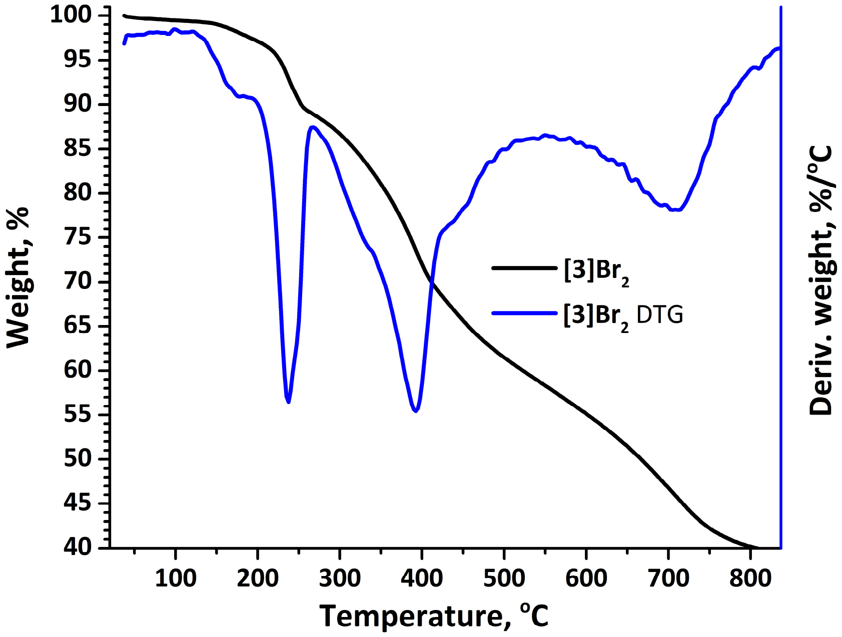

Figure S44. TGA and DTG curves of **[3]Br₂**. Heating rates of 10 ^{0}C·min^{-1}.

X-ray powder diffraction analysis of reaction mixture

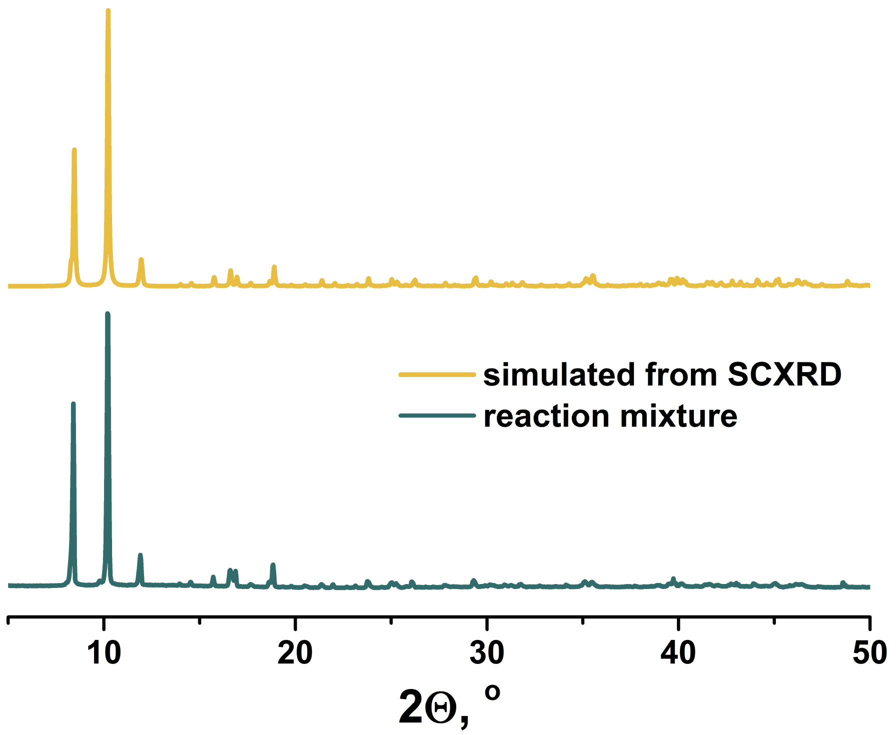

Figure S45. XRPD pattern of reaction mixture in comparison with calculated one from the SCXRD data for **[1^{red}]Br**.

Crystal structure data

Table S4. Selected crystallographic parameters of the single-crystal X-ray diffraction structural analysis for $[\{Mo_5(\mu_3\text{-}S)^i_4(\mu_4\text{-}S)^i(\mu\text{-}pz)^i_4\}(pzH)^t_5]Br\ pzH\cdot H_2O$ (**[1^{red}]Br**), $[\{Mo_5(\mu_3\text{-}S)^i_4(\mu_4\text{-}S)^i(\mu\text{-}pz)^i_4\}(pzH)^t_5][Mo_6I_{14}]\cdot 4CH_3CN$ (**[1^{ox}][Mo$_6$I$_{14}$]**), $[\{Mo_5(\mu_3\text{-}S)^i_4(\mu_4\text{-}S)^i(\mu\text{-}pz)^i_4\}(pzH)^{bs}_4Br^a]Br\cdot 2.5H_2O\cdot 0.5DMF$ (**[2]Br**$\cdot 2.5H_2O\cdot 0.5DMF$), $[\{Mo_5(\mu_3\text{-}S)^i_4(\mu_4\text{-}S)^i(\mu\text{-}Br\text{-}pz)^i_4\}(pzH)^t]Br_2\cdot CH_3CN\cdot Et_2O$ (**[3]Br$_2$**$\cdot CH_3CN\cdot Et_2O$), $[\{Mo_5(\mu_3\text{-}S)^i_4(\mu_4\text{-}S)^i(\mu\text{-}4\text{-}Br\text{-}pz)^i_4\}(pzH)^{bs}_4Br^a]Br\cdot 1.25H_2O\cdot 2DMSO$ (**[4]Br**$\cdot 1.25H_2O\cdot 2DMSO$).

Compound	**[1^{red}]Br**	**[1^{ox}][Mo$_6$I$_{14}$]**	**[2]Br·2.5H$_2$O·0.5DMF**	**[3]Br$_2$·CH$_3$CN·Et$_2$O**	**[4]Br·1.25H$_2$O·2DMSO**
Empirical formula	$C_{30}H_{38}BrMo_5N_{20}OS_5$	$C_{35}H_{44}I_{14}Mo_{11}N_{22}S_5$	$C_{25.5}H_{36.5}Br_2Mo_5N_{16.5}O_3S_5$	$C_{33}H_{41}Br_6Mo_5N_{19}OS_5$	$C_{28}H_{36.5}Br_6Mo_5N_{16}O_{3.25}S_7$
Formula weight	1414.71	3765.16	1422.03	1839.31	1832.81
Temperature, K	150(2)	150(2)	150(2)	150(2)	150(2)
Crystal system	Orthorhombic	Monoclinic	Monoclinic	Monoclinic	Monoclinic
Space group	*P* ccn	*C* 2/c	*C* 2	*P* 2_1/n	*P* 2_1/m
a, Å	14.7541(3)	15.4321(9)	14.6680(16)	12.1687(5)	12.4399(4)
b, Å	14.8128(5)	15.6714(9)	23.7909(16)	15.4833(6)	20.9936(7)
c, Å	21.3266(7)	33.2330(17)	13.6723(11)	30.8952(12)	13.9325(5)
α, °	90	90	90	90	90
β, °	90	92.317(2)	90.281(4)	94.8520(10)	107.891(2)
γ, °	90	90	90	90	90
V, Å^3	4660.9(2)	8030.6(8)	4771.1(7)	5800.2(4)	3462.6(2)
Z	4	4	4	4	2
ρ_{calc}, g/cm^3	2.016	3.114	1.980	2.106	1.758
μ, mm^{-1}	2.442	7.209	3.219	5.412	4.591
F (000)	2764	6768	2756	3520	1749
Crystal size	0.18 × 0.14 × 0.08	0.06 × 0.02 × 0.02	0.115 × 0.10 × 0.006	0.05 × 0.04 × 0.004	0.16 × 0.09 × 0.065
2Θ range for data collection, °	2.170 to 27.507	1.935 to 27.516	2.204 to 27.540	1.472 to 30.560	1.536 to 28.733
Index ranges	$-11 \le h \le 18$<br>$-19 \le k \le 19$<br>$-27 \le l \le 27$	$-20 \le h \le 20$<br>$-20 \le k \le 20$<br>$-42 \le l \le 43$	$-19 \le h \le 17$<br>$-30 \le k \le 30$<br>$-17 \le l \le 17$	$-17 \le h \le 17$<br>$-22 \le k \le 22$<br>$-44 \le l \le 43$	$-16 \le h \le 16$<br>$-28 \le k \le 28$<br>$-18 \le l \le 18$
Reflections collected	34739	47817	23934	74634	40306
Independent reflections	5324 [R_{int} = 0.0571]	9222 [R_{int} = 0.0547]	10881 [R_{int} = 0.0604]	17726 [R_{int} = 0.1217]	9171 [R_{int} = 0.0496]
Data/restraints/parameters	5324/27/305	9222/12/396	10881/61/580	17726/0/626	9171/18/358
Goodness-of-fit on F^2	1.022	1.009	1.075	1.029	1.050
R_1 / wR_2(I > 2σ(I))	0.0294/0.0586	0.0301/0.0551	0.0600/0.1559	0.0778/0.1774	0.0733/0.2064
R_1 / wR_1 (all data)	0.0412/0.0611	0.0465/0.0596	0.0912/0.1707	0.1485/0.1977	0.0972/0.2169
$\Delta\rho_{max}/\Delta\rho_{min}$ (e·Å^{-3})	1.135/–0.789	0.935/–0.930	1.914/–1.316	2.246/–1.163	2.150/–1.054
